# Platelet function is modified by common sequence variation in megakaryocyte super enhancers

**DOI:** 10.1038/ncomms16058

**Published:** 2017-07-13

**Authors:** Romina Petersen, John J. Lambourne, Biola M. Javierre, Luigi Grassi, Roman Kreuzhuber, Dace Ruklisa, Isabel M. Rosa, Ana R. Tomé, Heather Elding, Johanna P. van Geffen, Tao Jiang, Samantha Farrow, Jonathan Cairns, Abeer M. Al-Subaie, Sofie Ashford, Antony Attwood, Joana Batista, Heleen Bouman, Frances Burden, Fizzah A. Choudry, Laura Clarke, Paul Flicek, Stephen F. Garner, Matthias Haimel, Carly Kempster, Vasileios Ladopoulos, An-Sofie Lenaerts, Paulina M. Materek, Harriet McKinney, Stuart Meacham, Daniel Mead, Magdolna Nagy, Christopher J. Penkett, Augusto Rendon, Denis Seyres, Benjamin Sun, Salih Tuna, Marie-Elise van der Weide, Steven W. Wingett, Joost H. Martens, Oliver Stegle, Sylvia Richardson, Ludovic Vallier, David J. Roberts, Kathleen Freson, Lorenz Wernisch, Hendrik G. Stunnenberg, John Danesh, Peter Fraser, Nicole Soranzo, Adam S. Butterworth, Johan W. Heemskerk, Ernest Turro, Mikhail Spivakov, Willem H. Ouwehand, William J. Astle, Kate Downes, Myrto Kostadima, Mattia Frontini

**Affiliations:** 1 Department of Haematology, University of Cambridge, Cambridge Biomedical Campus, Cambridge CB2 0PT, UK; 2 National Health Service Blood and Transplant (NHSBT), Cambridge Biomedical Campus, Cambridge CB2 0PT, UK; 3 Nuclear Dynamics Programme, The Babraham Institute, Babraham Research Campus, Cambridge CB22 3AT, UK; 4 NIHR BioResource-Rare Diseases, University of Cambridge, Cambridge Biomedical Campus, Cambridge CB2 0QQ, UK; 5 European Molecular Biology Laboratory, European Bioinformatics Institute, Wellcome Genome Campus, Hinxton, Cambridge CB10 1SD, UK; 6 Medical Research Council Biostatistics Unit, University of Cambridge, Forvie Site, Cambridge Biomedical Campus, Cambridge CB2 0SR, UK; 7 Department of Human Genetics, The Wellcome Trust Sanger Institute, Wellcome Trust Genome Campus, Hinxton, Cambridge CB10 1SA, UK; 8 Strangeways Research Laboratory, The National Institute for Health Research (NIHR) Blood and Transplant Unit in Donor Health and Genomics at the University of Cambridge, University of Cambridge, Cambridge CB1 8RN, UK; 9 Department of Biochemistry, Cardiovascular Research Institute Maastricht, Maastricht University, PO Box 616, 6200 MD Maastricht, The Netherlands; 10 Strangeways Research Laboratory, MRC/British Heart Foundation (BHF) Cardiovascular Epidemiology Unit, Department of Public Health and Primary Care, University of Cambridge, Cambridge CB1 8RN, UK; 11 Department of Clinical Laboratory Sciences, College of Applied Medical Sciences, University of Dammam, P.O. Box 1982, Dammam 31441, Saudi Arabia; 12 Department of Medicine, University of Cambridge, Cambridge Biomedical Campus, Cambridge CB2 0QQ, UK; 13 NIHR Cambridge Biomedical Research Centre hIPSC Core Facility, Department of Surgery, University of Cambridge, Cambridge Biomedical Campus, Cambridge CB2 0SZ, UK; 14 Wellcome Trust and MRC Cambridge Stem Cell Institute, Department of Surgery, University of Cambridge, Cambridge Biomedical Campus, Cambridge CB2 0SZ, UK; 15 Genomics England Limited, Queen Mary University of London, Dawson Hall, London EC1M 6BQ, UK; 16 Faculty of Science, Department of Molecular Biology, Radboud University, 6525GA Nijmegen, The Netherlands; 17 The Wellcome Trust Sanger Institute, Wellcome Trust Genome Campus, Hinxton, Cambridge CB10 1SA, UK; 18 Radcliffe Department of Medicine, John Radcliffe Hospital, University of Oxford, Headington, Oxford OX9 3DU, UK; 19 Department of Haematology, Churchill Hospital, Headington, Oxford OX3 7LE, UK; 20 NHSBT, John Radcliffe Hospital, Headington, Oxford OX3 9BQ, UK; 21 Department of Cardiovascular Sciences, Center for Molecular and Vascular Biology, University of Leuven, Leuven 3000, Belgium; 22 BHF Centre of Excellence, Division of Cardiovascular Medicine, Addenbrooke’s Hospital, Cambridge Biomedical Campus, Cambridge CB2 0QQ, UK; 23 Department of Biological Science, Florida State University, Tallahassee, Florida 32303, USA

## Abstract

Linking non-coding genetic variants associated with the risk of diseases or disease-relevant traits to target genes is a crucial step to realize GWAS potential in the introduction of precision medicine. Here we set out to determine the mechanisms underpinning variant association with platelet quantitative traits using cell type-matched epigenomic data and promoter long-range interactions. We identify potential regulatory functions for 423 of 565 (75%) non-coding variants associated with platelet traits and we demonstrate, through *ex vivo* and proof of principle genome editing validation, that variants in super enhancers play an important role in controlling archetypical platelet functions.

## Abstract (web summary)

Numerous genetic variants, including those located in the non-coding regions of the genome, are known to be associated with blood cells traits. Here, Frontini and colleagues investigate their potential regulatory functions using epigenomic data and promoter long-range interactions.

Blood cells traits such as counts and mean cellular volumes are highly heritable and can be readily measured using hematology analysers as part of a complete blood count (CBC). We identified, by genome-wide association study (GWAS), 2,706 independent sentinel variants associated with 36 CBC-measured traits of blood cells^1^. Of these variants, 674 are associated with the count, the mean volume, the width of the volume distribution or the mass (also known as crit, count × mean volume) of platelets (CBC-P hereafter). Platelets are the smallest cells of the blood and their functions are to initiate repair at sites of vascular injury and to maintain haemostasis; furthermore, they are implicated in the aetiologies of myocardial infarction and stroke, among the leading causes of morbidity and mortality worldwide.

Platelets and red cells are formed by megakaryocytes (MKs) and erythroblasts (EBs), which originate through a stepwise differentiation of the haematopoietic stem cell (HSC)^2^. Red cell production depends on iron homeostasis^3^ and oxygen sensing^3^, whereas platelet production is controlled by a negative feedback loop. This is based on circulating thrombopoietin level, which is directly linked to platelet count, because platelets bind and degrade thrombopoietin via its receptor myeloproliferative leukemia protein (MPL) on their surface^4^. Platelets and MKs therefore provide an excellent model to link trait-associated variants to the genes they may regulate.

The majority of CBC-P-associated variants are located in the non-coding genomic space and therefore it remains challenging to explain their mechanism of action. GWAS signals are enriched in enhancer elements^5^. Enhancers function through chromatin loops, physically connecting them with the promoters of their target gene(s)^6^,^7^ often bypassing the nearest gene^8^. Here, to determine the mechanisms underpinning variant association with platelet quantitative traits, we integrate MK and EB promoter capture Hi-C (PCHi-C)^9^, a core set of histone modifications and CCCTC-binding factor (CTCF)-binding data generated as part of this and the BLUEPRINT consortium studies^10^,^11^. We propose a mapping strategy able to identify potential regulatory functions for 423 of 565 (75%) of CBC-P non-coding variants. Moreover, we provide examples of the effect of common variation on transcriptional mechanisms, which reveal that CBC-P in MK super enhancers (SEs) modify platelet functions.

## Results

### MK and EB open chromatin dynamics

Most associations between variants and traits are limited to a single type of blood cell; for example, only 41 of the 674 (6.1%) CBC-P-associated sentinel variants are pleiotropic, that is, also associated with red cell traits^1^. Earlier studies suggest that this restriction of associations to a single-cell lineage is in part explained by associated variants being located in cell-type-specific open chromatin elements^12^,^13^,^14^,^15^.

**Figure 1:**
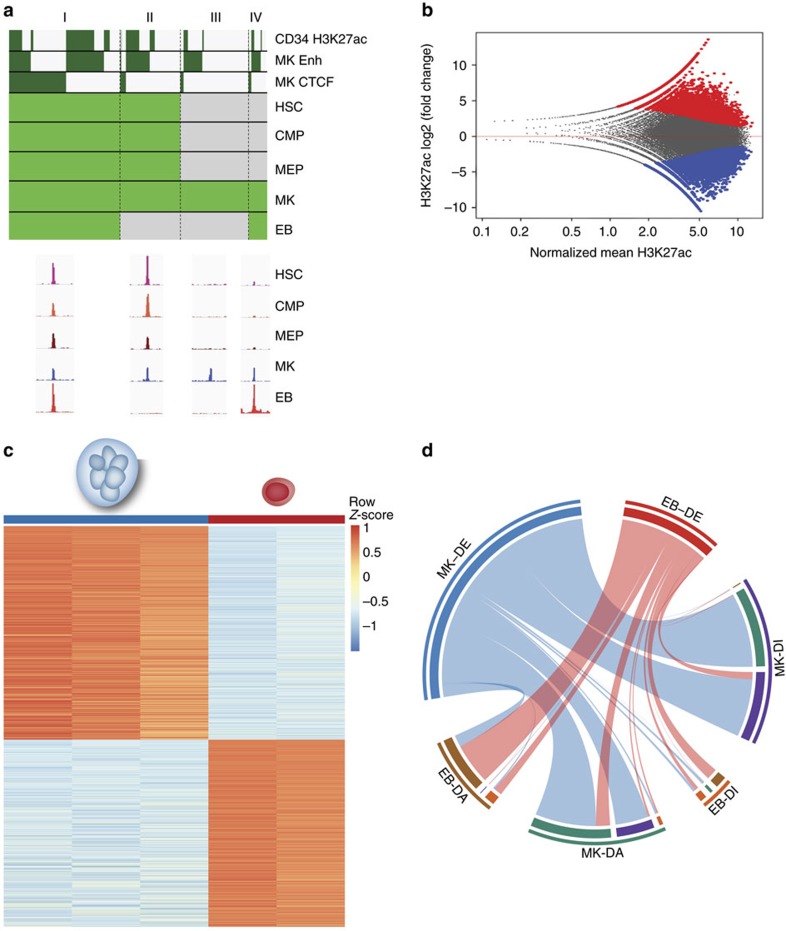
Unique three-dimensional regulatory landscapes define megakaryopoiesis and erythropoiesis. (**a**) Top panel, MK ATAC-seq peak (126,428) dynamics from HSCs through CMPs and MEPs, as well as EBs open chromatin as determined by DNase-seq (light green and grey, open and closed chromatin, respectively). H3K27ac in CD34+ haematopoietic stem and progenitor cells (HSPCs, data from ROADMAP), enhancer regions (Enh) and CTCF binding sites in MKs have been added for comparison (dark green, present). Categories: (I) Open chromatin regions present in all five cell types. In MKs 24,318/47,502 (51.2%) of ATAC-seq peaks were CTCF-binding sites and 25,548/47,502 (53.8%) of these were enhancers. (II) Open chromatin regions present from HSCs to MKs, but absent from EBs. (III) Open chromatin regions present either only in MKs or (IV) only in MKs and EBs. Bottom panel, representative examples of open chromatin peaks for the four categories. (**b**) Categorization of elements based on differences in H3K27ac signal intensities: black, nonsignificantly different (*n*=∼57,000); blue and red, significantly higher in MKs (*n*=6,810) and EBs (*n*=5,237), respectively. (**c**) Heatmap of 1,546 genes differentially expressed (DE) in RNA-seq analysis of MKs (left) and EBs (right). (**d**) Circular plot representing the interactions between DE genes (MK-DE, light blue; EB-DE, red), differentially acetylated (DA) elements (MK-DA, green; EB-DA, brown) and differentially interacting (DI) elements (MK-DI, dark blue; EB-DI, orange) on the outer arcs. Inner arc colours follow the same colour scheme and indicate overlap of attributes for these categories. Connections reflect a concordance of fold changes: DE genes in MKs tend to interact with regions specifically acetylated in MKs compared with EBs and vice versa.

To further characterize the lineage restriction of the CBC-P associations we generated open chromatin maps for the different stages of MK differentiation: HSCs, common myeloid progenitors (CMPs), MK–EB progenitors (MEPs) and MKs, as well as EBs (Supplementary Fig. 1). We found that 87.7% (110,844 of 126,428) of open chromatin regions in MKs fell into four categories (Fig. 1a, Supplementary Fig. 2 for EBs and Supplementary Data 1). The first (category I) contained open chromatin regions present from HSCs through to MKs and EBs. Category II comprised elements that were open throughout differentiation, but were closed in EBs, whereas categories III and IV consisted of elements that opened during the final stage of differentiation, either only in MKs (III) or in both MKs and EBs (IV). To identify the genes regulated by these elements, we used PCHi-C data^16^ (Supplementary Fig. 3, Supplementary Table 1 and Supplementary Data 2). We experimentally determined the genomic loci occupied by CTCF, a structural protein involved in the establishment of DNA loops^17^, in MKs and EBs, and found that promoter-interacting fragments have higher density of bound CTCF than the rest of the genome (*P*<2.2 × 10^−16^, zero-inflated negative binomial test); this was the case both when CTCF peaks were located in open chromatin or outside open chromatin regions (in both cases, *P*<2.2 × 10^−16^, negative binomial test, Supplementary Table 2). Moreover, we found that open chromatin density is higher in promoter-interacting fragments (*P*<2.2 × 10^−16^, zero-inflated negative binomial test, Supplementary Table 2) as are chromatin modifications^16^.

Gene Ontology (GO) terms enrichment analysis for genes interacting with open chromatin elements in any of the four categories described above revealed terms related to platelet functions interspersed among more generic terms relating to cellular metabolism and processes (Supplementary Data 3), indicating that the key cellular functions of platelets and red cells are not controlled solely by elements activated late in differentiation (Categories III and IV). We investigated whether a more meaningful enrichment of GO terms could be observed by assigning function to the MK and EB genomes according to their epigenetic state. Analysis of the data generated by the BLUEPRINT consortium for six histone marks with the IDEAS^18^ chromatin segmentation algorithm showed that the majority of segments had the same epigenomic state in MKs and EBs (Supplementary Fig. 4). Less than 20% of the genomic space labelled as ‘enhancer’ in either MKs or EBs had a different state in the other cell type, with ‘weak enhancer’ being the most frequent state transition (Supplementary Fig. 4).

### MK and EB regulatory landscape

Considering these results, we further explored differences between MKs and EBs that could explain their distinct transcriptomes. To highlight possible differences in enhancers’ activity we compared the strength of H3K27ac signals between MKs and EBs, and identified just 12,047 (17.5%) elements that differed significantly, with 5,237 and 6,810 preferentially acetylated in MKs and EBs, respectively (twofold change, 0.05 false discovery rate; Fig. 1b and Supplementary Data 4). Analysis of BLUEPRINT RNA sequencing data identified 1,546 genes differentially expressed between MKs and EBs (Fig. 1c, estimated fold change >2, posterior probability for differential expression >0.5, Supplementary Data 5). We then analysed PCHi-C interaction data and found that enhancers with higher acetylation levels in MKs were enriched for interactions with MK upregulated genes (Fisher’s exact test, *P*<10^−16^; odds ratio (OR) of 3.3; Fig. 1d and Supplementary Fig. 5a). Similarly, we detected enrichment for differentially expressed genes in the promoter interactions with differential intensities between MKs and EBs (Fisher’s exact test, *P*<10^−16^; OR 3.9; Supplementary Fig. 5b). Interestingly, the differentially acetylated enhancers in either cell type are more frequently located in the proximity of other differentially acetylated enhancers than expected by chance (Fisher’s exact test, *P*<10^−16^; OR 7.3; Supplementary Fig. 5c).

### SEs define MK and EB cell identities

**Figure 2:**
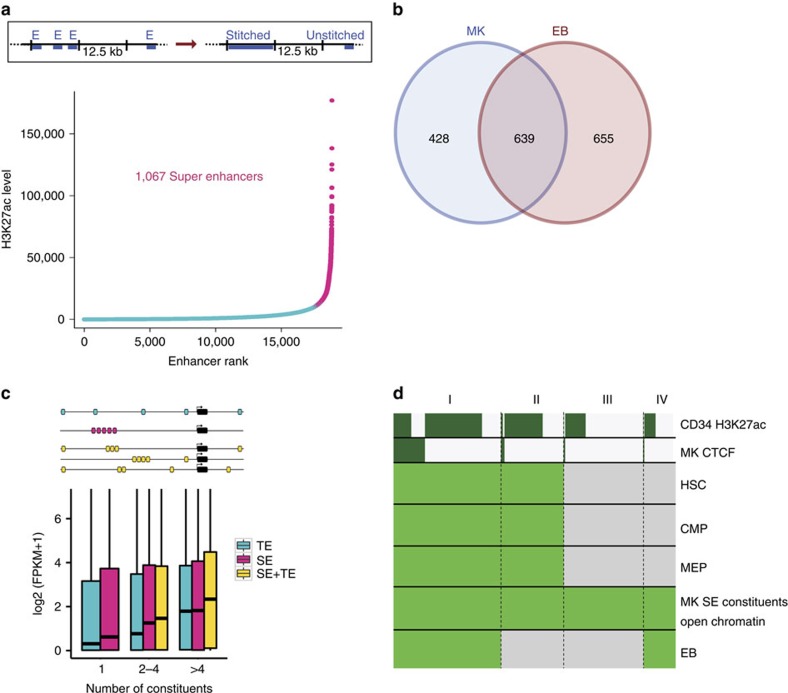
Identification of SEs their effects on gene expression and their opening dynamics. (**a**) Schematic of the stitching process to identify enhancer clusters and ranking based on H3K27ac signal intensities. (**b**) Overlap of SE sets in MKs and EBs. (**c**) Gene expression, in MKs, for genes connected to TEs only (blue), SE constituents only (pink), or a combination of TEs and SE constituents (yellow) (box plot: line indicates median, upper and lower box margins indicate first and third quartile). Top row of schematic shows a gene regulated by five TEs, second row shows a gene regulated by five SE constituents and the bottom rows show genes regulated by different combinations of five TEs and SE constituents. *P*-values for Wilcoxon test between different categories are in Supplementary Table 3. (**d**) Opening dynamics of MK SEs constituents during HSC differentiation. Open chromatin regions overlapping with MK SE constituents in HSCs, CMPs, MEPs and EBs. H3K27ac in CD34+ haematopoietic stem and progenitor cells (HSPCs) and CTCF-binding sites in MKs added for comparison (colour legend as in Fig. 1a).

To expand on this observation of co-location of differentially acetylated elements, we defined SEs in both MKs and EBs, as these are considered the drivers of cell type-specific gene expression. SEs are composed of physically proximal enhancers (constituents) and have higher than usual H3K27 acetylation and density of bound transcription factors^19^,^20^,^21^. Using the analytical approach described in Whyte *et al*.^20^, albeit not free from controversy especially for those enhancers close to the threshold^22^, we identified 1,067 and 1,287 SEs in MKs and EBs, respectively, 639 being shared (Fig. 2a,b, Supplementary Fig. 6 and Supplementary Data 6). The remaining enhancers with H3K27ac signals below the threshold (Fig. 2a, Methods) were called other enhancers and their constituents typical enhancers (TEs). We categorized genes according to the number of interacting enhancers and observed that genes linked to SE constituents had higher median expression than genes linked to TEs, across the categories and independently of the constituent number (Fig. 2c, Supplementary Fig. 7a–c and Supplementary Table 3). To determine when SEs in MKs become activated, we used open chromatin data for the five populations of blood progenitor cells and categorized the SE constituent opening patterns during differentiation from HSCs to MKs and EBs. This analysis showed that half of the SE constituents in MKs overlapped open chromatin regions in HSCs, two-thirds of which already had an H3K27ac mark in CD34+ haematopoietic stem and progenitor cells (Fig. 2d and Supplementary Data 7). However, only a small fraction of SEs (24/1,067 and 45/1,287 in MKs and EBs, respectively) had all their constituent enhancers open in HSCs and at the level of CMPs and MEPs (Fig. 2d and Supplementary Fig. 7d,e). Constituents that are in category I were also found to have a higher number of PCHi-C interactions when compared with each of the other categories (Wilcoxon test results in Supplementary Fig. 7f,g legend). Thus, the control of genes determining the distinct functional identities of MKs and EBs seems to be achieved by the opening of just 2,125 (17.9%) and 2,263 (16.4%) of SE constituents in MKs and EBs, respectively, at the final stage of differentiation (Supplementary Data 7).

### Mapping platelet traits variants with functional genomics

**Figure 3:**
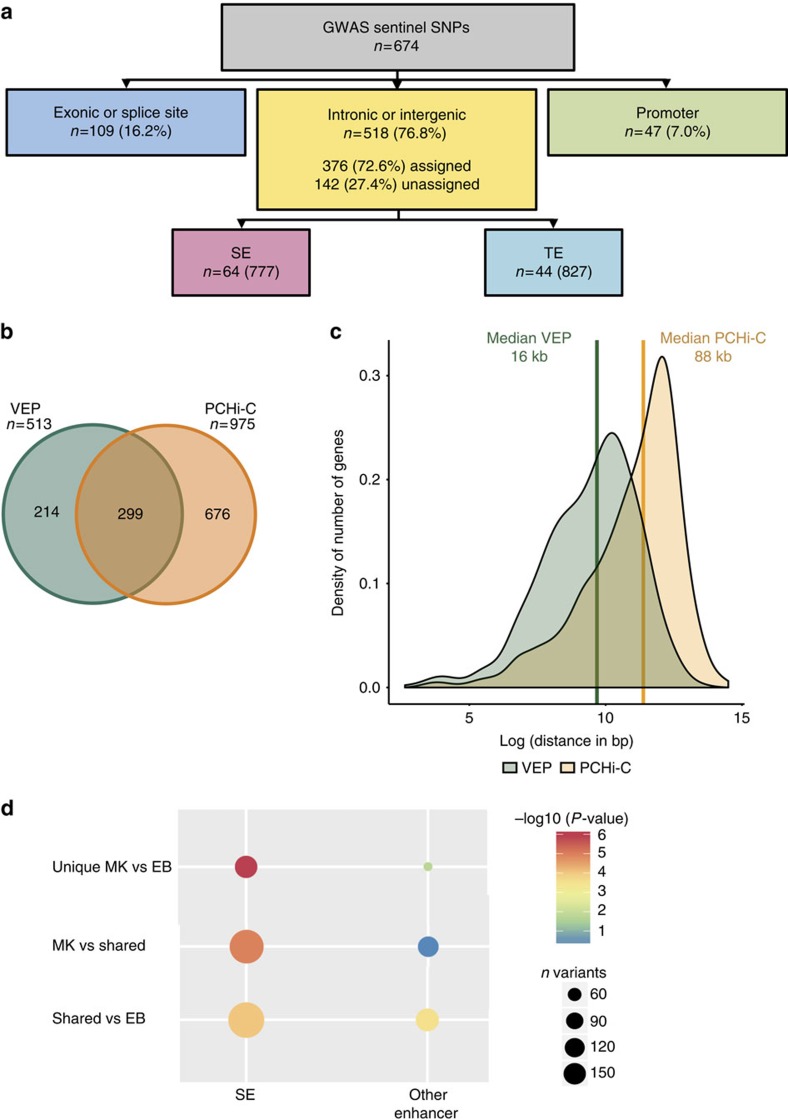
GWAS non-coding sentinel variants associated with platelet traits are enriched in SEs of MKs. (**a**) Categorization of sentinel variants associated with CBC-P (count, mean volume, volume width distribution and platelet crit (mean volume × count)) by location; exonic or splice site (light blue), intronic or intergenic (yellow) and promoter (green). Number of intronic or intergenic SNPs localized to SE constituents and TEs, detailed description of annotation in Supplementary Fig. 8a. (**b**) Venn diagram showing the overlap of the sets of genes to which the CBC-P-associated variants were assigned by variant effect predictor (VEP, green) and by the analysis reported in this study (orange). (**c**) Density distribution of the genomic distance between a CBC-P sentinel SNP and the transcriptional start site (TSS) of the gene it has been assigned to by VEP (green) and the approach used in this study (orange). For genes with several TSSs, the mean position of all TSSs was used. (**d**) *P*-values characterizing the significance of difference between the prevalence of CBC-P versus CBC-red cell trait-associated non-coding sentinel variants within SE and other enhancers. All *P*-values are based on a permutation test involving 999,999 simulations of locations of significantly associated sentinel variants. Each dot corresponds to a comparison of two categories of enhancers—the cell types of both enhancers are indicated on *y* axis and the enhancer type is denoted on *x* axis. The surface area of each dot is proportional to the number of significant association signals either for CBC-P or CBC-red cell traits residing within either of the two enhancers being compared (pleiotropic variants are not counted). Number of variants tested for each category available in Supplementary Table 10.

Our integrative analysis focused on 674 unique sentinel variants associated with the CBC-P traits identified in our recent GWAS in 173,480 individuals^1^. The majority (*n*=565, 84%) of variants are non-coding (intronic, intergenic or located in a promoter); 47 and 141 variants overlapped a promoter or enhancer in MKs, respectively (Fig. 3a, Supplementary Fig. 8a and Supplementary Data 8). Another 980 variants, from a set of 6,176 single-nucleotide polymorphisms (SNPs) in linkage disequilibrium (LD; *r*^2^>0.8; whole-genome sequencing data of 6,687 NIHR BioResource—Rare Diseases samples) with sentinel variants, were also located in enhancers (Fig. 3a). Interestingly, we observed a fivefold enrichment of CBC-P sentinel variants located in SE constituents relative to TEs in MKs (Fisher’s exact test, *P*<2.2 × 10^−16^, OR 5.1). The successful assignment of the coding and 75% of the non-coding CBC-P-associated variants identified a set of 975 genes (Fig. 3b and Supplementary Fig. 8b depicts a Cytoscape displayed protein–protein interaction network of 4,235 nodes and 18,550 edges, which was generated by using 781 of the 975 genes as baits to retrieve interactors). Only 205 variants (30%) were assigned solely to the nearest gene, whereas 123 variants (18%) were assigned to the nearest gene and additional genes, and 204 (30%) were linked to distal genes. Indeed, the median distance of the new set of assigned genes to associated variants was 88 kb compared with a median of 16 kb for the gene set inferred by the coordinate-based approach still widely used for the functional annotation of GWAS variants^1^ (Fig. 3c). The importance of having data on long-range interaction between promoters and regulatory elements in a relevant cell type was further illustrated by circular genomic permutation analysis^23^ using the SEs and other enhancers in MKs and EBs, respectively. This analysis showed that CBC-P-associated variants, but not red cell ones, were more likely to be located in MK-specific SEs and were less likely to be found in other enhancers or in shared and EB-specific SEs (Fig. 3d and Supplementary Table 4). The circular permutation analysis also provided orthogonal evidence of qualitative differences between the SE and TE.

Using interaction data, we linked the 1,067 SEs in MKs to 3,339 genes; SE-connected genes were enriched for the GO terms haemostasis, degranulation and coagulation, which are archetypical for platelet function and thrombus formation (Supplementary Data 6). These enrichments were even more evident when only protein-coding genes connected to MK SEs that harbour a CBC-P sentinel variant or proxy were considered, as no other terms were found (Supplementary Fig. 8c and Supplementary Data 9). To determine whether CBC-P-associated loci also modulate the thrombotic function of platelets we tested the CBC-P sentinel variants for association with quantitative responses of platelets to activation by ADP and the collagen mimetic CRP-XL in a cohort of just more than 1,200 genome-wide typed healthy subjects^24^. Four CBC-P sentinel variants, rs1613662 (*GP6*), rs12041331 (*PEAR1*), rs3557 (*FCER1G*) and rs1354034 (*ARHGEF3*) were associated with at least one platelet function trait at *P*<5 × 10^−7^.

### SE variation and platelet functions

**Figure 4:**
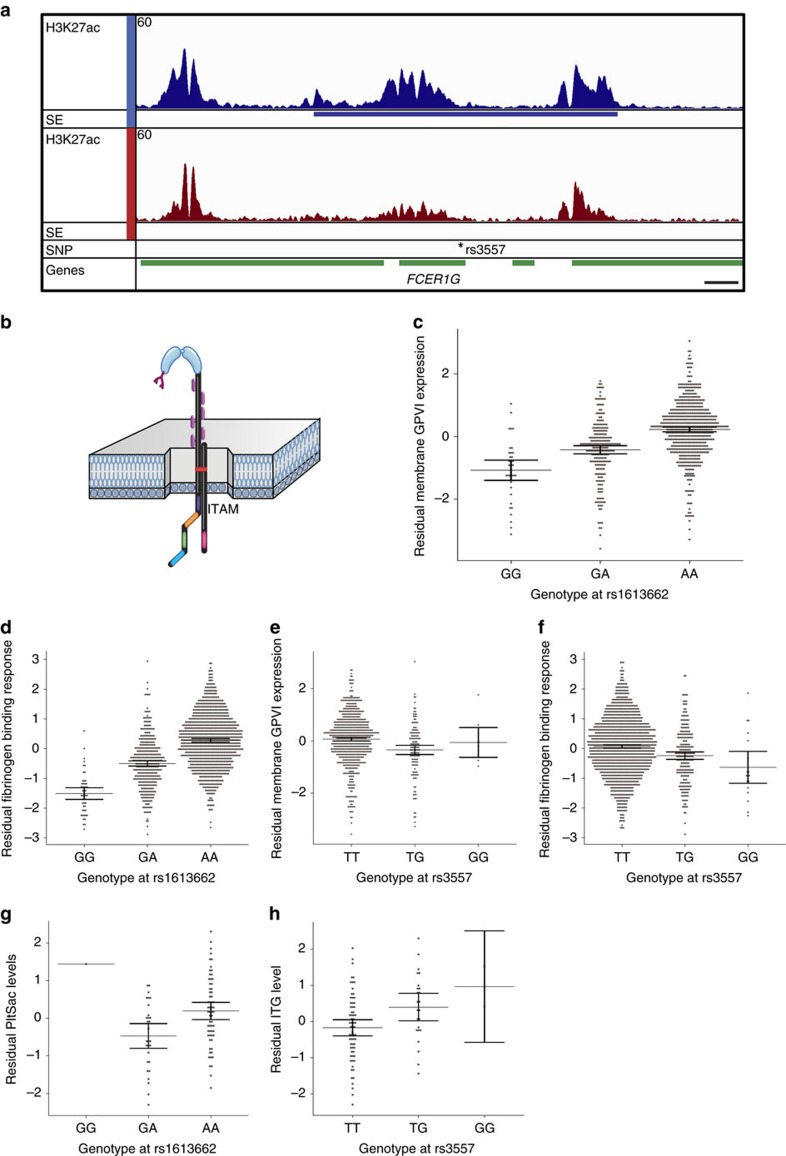
Association between SE-localized sentinel variant rs3557 and thrombus phenotypes. (**a**) Chr1 1q23.3 locus view comprising *FCER1G* and three other genes. From top to bottom: H3K27ac signal track and SE location in MKs (blue) and EBs (red); *position of sentinel variant rs3557 and genes in green. Scale bar in bottom right corner represents 2 kb. Maximum read signal scale 60 for each track. (**b**) Schematic representation of the glycoprotein (GP)VI/Fc receptor *γ*-chain signalling receptor complex for collagen on platelets. (**c**–**h**) Associations of genotypes of rs1613662 and rs3557 with the residuals of platelet function phenotypes, after adjustment for covariates. Dots show distribution of the phenotypic residuals; central lines show genotype-specific mean estimates and whiskers represent 95% confidence intervals. (**c**,**e**) Associations with platelet membrane level of GPVI after linear adjustement for the interaction of logged mean platelet volume and sex (rs1613662: GG=36, GA=221, AA=587, likelihood ratio additive *P*=1.6 × 10^−27^; rs3557, TT=696, TG=139, GG=9, likelihood ratio additive *P*=4.6 × 10^−5^). (**d**,**f**) Associations of fibrinogen binding to integrin αIIbβ3 after platelet activation with CRP-XL, adjusted for sex (rs1613662: GG=49, GA=381, AA=992, likelihood ratio additive *P*=1.6 × 10^−7^; rs3557, TT=1,175, TG=229, GG=18, likelihood ratio additive *P*=4.6 × 10^−72^). (**g**,**h**) Associations for rs1613662 and rs3557 with thrombus formation upon flowing whole blood over collagen III in microchambers, measured by quantile-normalized sex-adjusted platelet surface area coverage (PltSac; GG=1, GA=29, AA=63, likelihood ratio additive *P*=1.8 × 10^−2^) and quantile-normalized sex-adjusted activation of integrin αIIbβ3 (ITG; TT=67, TG=24, GG=2, likelihood ratio additive *P*=3.4 × 10^−3^), respectively.

The variant rs3557 is located in a SE interacting with the promoter of *FCER1G*, the gene encoding the *γ*-chain of the Fc receptor for IgE (Fig. 4a). This *γ*-chain also anchors the collagen signalling receptor glycoprotein (GP)VI (encoded by *GP6*) in the membrane of platelets (Fig. 4b). Here we replicate in a larger number of samples our earlier findings^24^ that subjects carrying the minor allele of the non-synonymous variant rs1613662 in *GP6* have lower levels of membrane GPVI and a concomitant reduced functional response of their platelets to the GPVI-specific ligand CRP-XL (Fig. 4c,d). We reasoned that, because of the functional association of GPVI and the *γ*-chain, variant rs3557 might also modify GPVI abundance and GPVI downstream signalling events. Indeed, when we tested these associations we observed that platelets of subjects carrying the minor allele of the SE-located variant rs3557 have lower average GPVI levels and reduced average αIIbβ3 integrin levels upon activation with CRP-XL (Fig. 4e,f). To explore this further, we examined thrombus formation under more physiological conditions (Supplementary Table 5). Platelets become activated by collagen released from a ruptured plaque, whilst being exposed to high shear. These conditions can be mimicked *ex vivo* by flowing whole blood over collagen-coated surfaces in microchambers^25^. As expected, the blood from subjects carrying the minor allele of rs1613662 (*GP6*) formed thrombi to a lesser extent than the blood from subjects lacking the minor allele (Fig. 4g). Unexpectedly, the association of rs3557 (*FCER1G)* with platelet activation by collagen III was of opposing direction compared with the effect of the variant in the platelet activation test with CRP-XL under static conditions (*P*=4.8 × 10^−4^; Fig. 4h). The opposite direction of the effects is best explained by the differences between the synthetic collagen mimetic CRP-XL, which only interacts with platelet GPVI versus collagen III, which does in addition to GPVI also engages integrin αIIbβ1 and GPIbα^26^.

**Figure 5:**
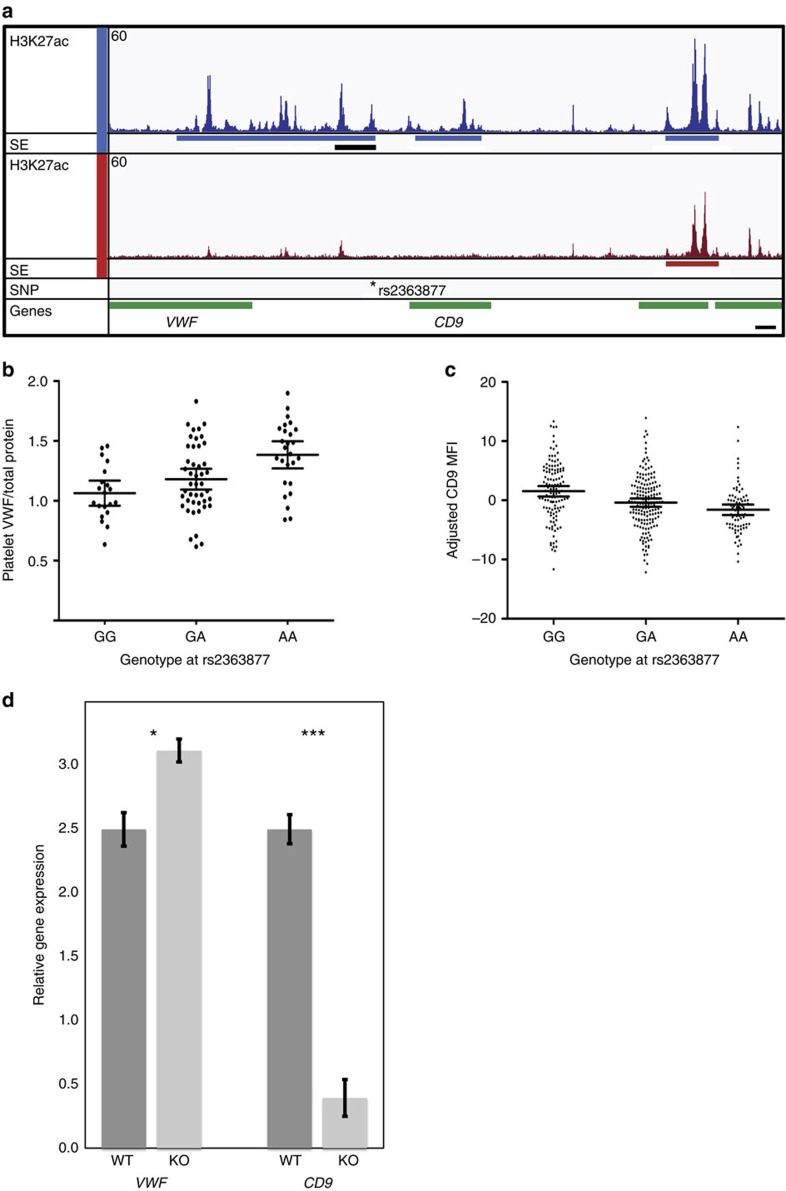
Effect of the SE-localized platelet trait associated sentinel variant rs2363877 on VWF and CD9 protein abundance. (**a**) Chr12p13.31 locus view comprising *VWF*, *CD9* and two other genes. From top to bottom: H3K27ac signal track and SE locations in MKs (blue) and EBs (red). Region of SE deleted by genome-editing (black); positions of sentinel variant rs2363877(*) and genes (green). Scale bar in bottom right corner represents 10 kb. Maximum read signal scale 60 for each track. (**b**,**c**) Associations of variant rs2363877 with (**b**) concentration of VWF in platelets (*Y* axis ng μl^−1^ normalized against total protein content; for subjects of genotypes: GG, *n*=20; GA, *n*=47; AA, *n*=26; likelihood ratio, *P*=10.0 × 10^−5^) and (**c**) CD9 abundance on platelet surface (*y* axis mean fluorescence intensity (MFI) adjusted for mean platelet volume; for subjects of genotypes: GG, *n*=122; GA, *n*=165; AA, *n*=78; likelihood ratio, *P*=1.3 × 10^−6^). Lines indicate mean, whiskers indicate 95% confidence interval. (**d**) Transcript levels of *VWF* and *CD9* in MKs obtained by forward programming of wild type and genome-edited pluripotent stem cells (*n*=3 biological replicates each in triplicate; error bars generated from s.e. calculated from delta Ct value across technical and biological replicates, Student’s *t*-test *P=2.2 × 10^−2^ and ***P=5.0 × 10^−4^).

We investigated a second example of a SE containing a CBC-P-associated variant chosen, because in high LD (*r*^2^>0.96, European ancestry subset of UK Biobank imputation data) with the mean platelet volume (MPV)- (rs4991925) and platelet distribution width (rs4290286)-associated variants identified in Astle *et al*.^1^. The SNP rs2363877 is located in a MK-specific SE interacting with the promoters of genes encoding the coagulation protein, Von Willebrand factor (VWF) and the tetraspanin CD9 (Fig. 5a). VWF tethers platelets to the vessel wall via its receptor GPIbα but VWF’s functional role in thrombus formation cannot be interrogated by the static platelet function tests and results from microchamber tests would have been confounded by VWF in plasma. We therefore used an alternative experimental approach to determine the possible effects of the sentinel variant rs2363877 on the regulation of the two genes. First, we identified associations of opposing direction with the levels of both VWF and CD9 proteins in platelets (Fig. 5b,c; Regression coefficient 0.163 (95% confidence interval=0.0821–0.243), *P*=10.0 × 10^−5^ and regression coefficient −1.1 (95% confidence interval =−2.3–1.0), *P*=1.3 × 10^−6^, respectively). Second, to characterize the mechanism by which the SE containing rs2363877 exerts its action on gene transcription, we used CRISPR/Cas9 to knock out part of the element in an induced pluripotent stem cell (iPSC) clone (Fig. 5a, black bar). In MKs obtained by forward programming^27^ of genome-edited iPSCs, we observed an effect on the transcript levels of both genes in the same direction as the minor allele of rs2363877, with a near-complete absence of the *CD9* transcript (Fig. 5d). The results of these experiments are compatible with the notion that the SE has both enhancing and repressive effects on the transcription of *CD9* and *VWF*, respectively. We assume that the different levels of VWF and CD9 proteins of platelets may modify the extent of thrombus formation and integrin signalling.

## Discussion

Altogether we found that just more than 32% of CBC-P-associated non-coding sentinel variants are located in enhancer elements or promoters of MKs and 423 (75%) of non-coding variants can now be linked with high confidence to the genes they regulate. The sentinel variants are enriched in MK SEs, which are often absent from EBs, thereby explaining in part the observation that most sentinel variants associated with platelet traits do not have an effect on red cell traits. Microchamber experiments and the use of genome-editing of iPSCs illustrate the role of SEs in the regulation of thrombus formation and the transcription of distant genes with important roles in haemostasis. Moreover, sentinel variants localized in SEs can have an effect on more than one gene highlighting the importance of genome conformation experiments to improve understanding of the molecular pathways underlying complex traits.

## Methods

### Purification of progenitor cell populations

Peripheral blood mononuclear cells were isolated using Ficoll-Paque gradients from apheresis filters, obtained from platelet donors after informed consent (A Blueprint of blood cells, REC 12/EE/0040, East of England-Hertfordshire Research Ethics committee). Progenitor cell populations were enriched by positive selection using CD34+ magnetic beads (130-046-702, Miltenyi) and purified by FACS sorting using a BD FACS Aria III. Progenitor cells were stained for flow cytometry analysis as previously described in Chen *et al*.^2^ and Supplementary Fig. 1 legend.

### Cord blood-derived MKs and EBs

Human cord blood was obtained after informed consent (A Blueprint of blood cells, REC 12/EE/0040, East of England-Hertfordshire Research Ethics committee), and MKs and EBs were generated through differentiation of CD34+ cord blood-derived cells as described in Chen *et al*.^2^.

### ATAC-seq libraries

Assay for transposase-accessible chromatin with high throughput sequencing (ATAC-seq) libraries were generated from freshly prepared cells using the protocol by Buenrostro *et al*.^28^. For MKs, 10^5^ cells were used with ten amplification cycles. For HSCs, CMPs and MEPs, 10^4^ cells were used with 12 amplification cycles. Libraries were quantified using a quantitative PCR (qPCR) Library Quantification Kit (Kapa Biosystems), pooled and sequenced with a 50 bp single-end protocol on an Illumina Hiseq 2,500.

### RNA-seq libraries

RNA sequencing (RNA-seq) libraries were generated by the BLUEPRINT Consortium. In brief, RNA was extracted from TRIzol preparations by phase-separation and precipitation. One microgram of DNase-treated RNA was used to generate ribosomal RNA-depleted libraries with a TruSeq Stranded Total RNA Library Prep Kit (with Ribo-Zero Human/Mouse/Rat, RS-122-2201, Illumina). Libraries were quantified using a qPCR Library Quantification Kit (Kapa Biosystems), pooled and sequenced using paired-end 76 bp sequencing on an Illumina Hiseq 2000.

### ChIP-seq libraries

Samples were fixed and prepared using the BLUEPRINT Consortium protocol. In brief, cells were fixed with 1% w/v formaldehyde for 10 min and quenched using 125 mM glycine before washing with PBS. Samples were sonicated using a Bioruptor (Diagenode), final SDS concentration of 0.1% w/v for 9 cycles of 30 s ‘on’ and 30 s ‘off’, and immunoprecipitated using an IP-Star Compact Automated System (Diagenode). For H3-specific antibodies the Auto-Histone ChIP-seq kit protein A (Diagenode) and for CTCF antibody the Auto iDeal ChIP-seq Kit for Transcription Factors (Diagenode) were used with Diagenode antibodies listed in Supplementary Table 6.

Immunoprecipitated and input DNA were reverse cross-linked (65 °C for 4 h), treated with RNase and Proteinase K (65 °C for 30 min). DNA was recovered with Concentrator 5 columns (Zymo) and prepared for sequencing using MicroPlex Library Preparation Kit v2 (Diagenode). Libraries analysed using High Sensitivity Bioanalyzer chips (5,067–4,626, Agilent), quantified using qPCR Library Quantification Kit (Kapa Biosystems), pooled and sequenced with a 50 bp single-end protocol on an Illumina Hiseq 2500.

### Platelet function analysis

This is an interim analysis of the Cambridge Platelet Function Cohort and the discrepancies between numbers of test for each agonist tested depend on when the assay was introduced. Platelet function testing and data analysis were performed as described in Jones *et al*.^24^ in up to 1,500 individuals by investigators blind to the tested subject genotype. For details please refer to Supplementary Information.

### VWF quantification in platelet lysates and plasma

VWF was quantified by ELISA; for details please refer to Supplementary Information.

### CD9 measurement on platelet surface

The surface expression of CD9 was measured, by using flow cytometry, in platelet rich plasma (PRP) of 365 healthy subjects, part of the Cambridge Platelet Function Cohort, by investigators blind to the subjects’ genotype. For details, please refer to Supplementary Information.

### VWF and CD9 genotype–phenotype associations

TaqMan assays (Applied Biosystems) were used to genotype whole-blood DNA extracted from the NIHR Cambridge BioResource volunteers using the manufacturer’s protocol. NHSBT blood donors were genotyped using Illumina genome wide typing array followed by imputation. To identify CD9 and VWF genotype–phenotype associations, we used linear regression models and tested for associations using likelihood ratio tests. Samples were excluded only if genotyping failed. A sample size of ∼100 individuals has been deemed sufficient to determine the extent of VWF and CD9 measured variation in platelet, given our assay sensitivities^24^,^25^ and rs2363877 allele frequency.

### Human iPSCs

A1ATD-1 iPSCs were cultured at 37 °C with 5% CO_2_ using Vitronectin (Life Technologies) treated plates and AE6 Media (DMEM/F12, Thermo Fisher), 0.05% w/v Sodium Bicarbonate (Thermo Fisher), 64.1 μg ml^−1^ L-Ascorbic acid 2-phosphate sesquimagnesium salt hydrate (Sigma), 1 × Insulin-Transferrin-Selenium (Thermo Fisher); supplemented with 15 ng ml^−1^ FGF2 (Cambridge Stem Cell Institute) and 15 ng ml^−1^ Activin A (Cambridge Stem Cell Institute).

### Genome editing of VWF-CD9 SE by CRISPR-Cas9

A 22 kb region located at one end of the VWF-CD9 SE 1 containing rs2363877 was knocked out (Fig. 5a, black bar). Single-guide RNAs (sgRNAs) were designed at either side of the target region (sgRNA1 and sgRNA2, Supplementary Table 7) using Protospacer WB software. Both strands were synthesized (IDT) with overhangs for ligation with BbsI sites of SpCas9-2A-Puro V2.0 (Addgene). To prepare SpCas9-2A-Puro V2.0, 1 μg was digested with 10 U of BbsI (NEB) for 1 h at 37 °C. Double-strand sgRNA1 and sgRNA2 oligonucleotides were ligated into the linearized plasmid using 600 U of T4 DNA ligase (NEB) for 1 h at 37 °C. Ligation products were transformed into competent α-Select Gold Efficiency Cells (Bioline) and plated on LB-agar ampicillin (100 μg ml^−1^) plates. Plasmids were verified by Sanger sequencing with U6-Forward Primer: 5′-GAGGGCCTATTTCCCATGATTCC-3′. Plasmid purification for nucleofection was performed using EndoFree Plasmid Maxi Kit (Qiagen) according to the manufacturer’s protocol. iPSCs were pre-treated with 10 μM ROCK inhibitor (Y-27632, Sigma) 4 hours before nucleofection, washed once with DPBS and incubated with Accutase (Thermo Fisher) for 5 minutes at 37 °C. Cells were dissociated into clumps of three to four cells and counted. Then 2 × 10^6^ cells were suspended in 100 μl of nucleofection P3 solution (Lonza) and electroporated with 8 μg of sgRNA1 and sgRNA2 expression vectors. Electroporation was performed using the 4D-Nucleofector System (Lonza) with the nucleofection program CA 137. Electroporated cells were plated onto 10 cm Vitronectin-coated plates in TeSR-E8 medium containing 10 μM ROCK inhibitor and incubated at 37 °C under 5% CO_2_. Puromycin selection (1 μg ml^−1^) commenced 24 h post nucleofection for 48 h. TeSR-E8 medium was changed daily. After 14 days single colonies were picked, expanded and genotyped (oligonucleotides described in Supplementary Table 8). Homozygous SE knockout (KO) iPSCs were generated at 15% efficiency.

### Forward programming of iPSC to MKs

A1ATD-1 iPSCs were forward programmed into MKs using the adherent cell protocol described Moreau *et al*.^27^. Cells were stained with CD41a-APC and CD42b-PE antibody conjugates (BD) and sorted using the FACS Aria Fusion (BD) FACS instrument.

### Gene expression in KO iPSCs using quantitative real-time PCR

Quantitative real-time PCR (qRT–PCR) was performed on complementary DNA generated from the forward programmed iPSC cell lines (A1ATD-1). The investigator performing the assay was aware of the genotype of the samples. Exon spanning oligonucleotides (Supplementary Table 9) were used to detect VWF, CD9 and the control gene GUSB.

qRT–PCR reactions used Brilliant II SYBR Green QPCR Master Mix (Agilent Technologies) and conditions: 95 °C, 5 min; 40 cycles of 95 °C, 30 s; 60 °C, 30 s and 72 °C, 30 s. Three iPSC lines of wild type and KO were tested (biological replicates) and qRT–PCR was performed in triplicate (technical replicates). Relative gene expression was presented as mean delta Ct against the reference and scaled so the wild-type expression levels of each gene were equal; error bars were generated from the s.e. calculated from the delta Ct values across technical and biological replicates. *t*-tests were used to analyse differences of the mean delta Ct values.

### Multimodular platelet activation in thrombus formation

Citrate-anticoagulated blood was used for multivariate platelet function analysis, using a microspot-based whole-blood microfluidics flow assay^25^,^29^. For details, please refer to Supplementary Information.

### RNA-seq analysis

Trim Galore 0.3.7 (http://www.bioinformatics.babraham.ac.uk/projects/trim_galore/) with parameters ‘-q 15 -s 3 --length 30 -e 0.05’ was used to trim PCR and sequencing adapters. Trimmed reads were aligned to the Ensembl v70 (ref. 30) human transcriptome with Bowtie 1.0.1 (ref. 31), with parameters ‘-a --best --strata -S -m 100 -X 500 --chunkmbs 256 --nofw -fr’. MMSEQ 1.0.8a (refs 32, 33), and was used with default parameters to quantify gene expression. Genes with posterior probability>0.5 (calculated by MMDIFF), absolute fold change >2 and fragments per kilobase of transcript per million mapped reads (FPKM) >1 in at least one of the two cell types were considered differentially expressed.

### ChIP-seq analysis

We applied the BLUEPRINT protocol for chromatin immunoprecipitation sequencing (ChIP-seq) data analysis: http://dcc.blueprint-epigenome.eu/#/md/chip_seq_grch37.

### CTCF peak calling

A cell-type-specific input was created by merging biological replicates into a single alignment file with ‘samtools merge’^34^,^35^. Peak calling was performed using MACS2 (ref. 36) (https://github.com/taoliu/MACS) after randomly down-sampling the input to the same number of reads in the corresponding sample and removing duplicates with PICARD tools (https://broadinstitute.github.io/picard/). To identify a set of reproducible CTCF peaks between the two EB replicates we used the irreproducible discovery rate analysis (https://sites.google.com/site/anshulkundaje/projects/idr). The maximum combined corrected *P*-value upon application of an irreproducible discovery rate threshold of 0.01 was used as a cutoff, to filter the CTCF MACS2 peaks called in the single-replicate MK sample. In total, we identified 38,326 CTCF peaks and 42,344 CTCF peaks in EB and MK, respectively.

### Genome segmentation

To identify genomic segments of recurring signal patterns across a set of six histone modifications (H3K4me1, H3K4me3, H3K9me3, H3K27ac, H3K27me3 and H3K36me3) in EBs and MKs, we used the genome segmentation algorithm IDEAS^18^. IDEAS jointly segments the genome across multiple cell types and infers the optimal number of distinct signal patterns, called states. We generated smoothened and normalized genome-wide signal per histone modification per cell type in bigwig format using align2rawsignal (https://github.com/akundaje/align2rawsignal) on two biological replicates. Then we used WiggleTools^37^ to count the mean number of reads per 200 bp bins across the genome. Finally, IDEAS identified 30 distinct states that were used to classify each 200 bp bin across genome in both cell types to one of these states. Each state was manually assigned a functional label, using as a guide the functional label assignment from Ernst *et al*.^38^. The 11 functional labels were as follows: inactive, heterochromatin, Polycomb repressed, transcribed, enhancer, bivalent enhancer, enhancer tail, promoter, weak promoter, bivalent promoter and promoter tail.

### CTCF enrichment in network elements

PCHi-C was performed using the restriction endonuclease *Hind*III^16^. Restriction fragments were overlapped with CTCF peaks in MKs and EBs. Restriction fragments overlapping ENCODE blacklisted regions (https://www.encodeproject.org/annotations/ENCSR636HFF/)) were removed. All remaining fragments were then overlapped with all connected baits as well as interacting regions (preys) in the respective cell types. A zero-inflated negative binomial regression on the peak counts per fragment was calculated on the number of interactions per fragment, accounting for the fragment length as logarithmic offset. The number of interactions was calculated for each fragment by counting to how many other fragments it was connected, using a CHiCAGO PCHi-C interaction score threshold of at least 5 (ref. 39).

### Open chromatin data analysis

EB DNase-seq data were obtained from Kellis *et al*.^40^ (GEO accession numbers GSE55579, GSM1339559 and GSM1339560). Raw Illumina DNase-seq reads were trimmed for quality using TrimGalore! v0.3.7 with a Phred score cut off of 15 (-q 15) (www.bioinformatics.babraham.ac.uk/projects/trim_galore/). MK, HSC, CMP and MEP ATAC-seq reads underwent quality and adapter trimming using TrimGalore! v0.3.7 with parameters -q 15 --stringency 3 -a 5′-CTGTCTCTTATACACATCTCTGA-3′. We followed the BLUEPRINT protocol for alignment of DNase-seq and ATAC-seq reads to GRCh37 using BWA and filtering of alignments (http://dcc.blueprint-epigenome.eu/#/md/dnase_seq_grch37) as well as for modelling fragment length with SPP^41^ and producing signal plots with align2rawsignal (http://dcc.blueprint-epigenome.eu/#/md/chip_seq_grch37) using the triweight smoothing method. Bedgraph files were converted to bigwig using bedGraphToBigWig^42^ (https://www.encodeproject.org/software/bedgraphtobigwig). Open chromatin peaks were called with F-seq^43^ with fragment size (-f) at 0 and the ‘s.d. threshold’ (-t) at 6. We removed peaks overlapping ENCODE blacklisted regions (https://www.encodeproject.org/annotations/ENCSR636HFF/) using bedtools v2.22.0 (ref. 44). For open chromatin data with two replicates, we called peaks separately, and retained and merged peaks present in both replicates (minimum overlap 1 bp) using bedtools merge.

### Open chromatin dynamics

We traced back the opening of MK ATAC-seq peaks (Fig. 1a, Supplementary Fig. 2a) and EB DNaseI-seq peaks (Supplementary Fig. 2b) by overlapping with ATAC-seq peaks called in HSCs, CMPs and MEPs (minimum overlap of 1 bp). CTCF labels were assigned based on overlap with CTCF peaks obtained in the corresponding cell type (MKs or EBs). Enhancer labels were assigned by overlapping open chromatin peaks±500 bp (to account for the shift between the open chromatin signal and the H3K27ac signal) with enhancers in MK or EB as identified by genome segmentation.

To determine which peaks had an H3K27ac signature in CD34+ cells, we used the consolidated epigenome file for H3K27ac and the corresponding input from ROADMAP Epigenomics (http://egg2.wustl.edu/roadmap/web_portal/processed_data.html). We converted the tagAlign files to bam files with bedtools v2.22.0, bedToBam and called peaks using MACS2 with the same parameters as used for CTCF peak calling. We overlapped open chromatin peaks±500 bp with the CD34+ H3K27ac peaks.

### Defining SEs

SEs in MKs and EBs were called based on regions identified as enhancers in the IDEAS genome segmentation (71,477 and 71,406 regions in MKs and EBs, respectively). We removed regions overlapping promoter, weak promoter and bivalent promoter states±1 kb to avoid confounding of enhancer and promoter H3K27ac signals. The remaining 52,929 enhancers for MKs and 54,944 enhancers for EBs were stitched together, if enhancers were within 12.5 kb, using ROSE (Fig. 2a, top panel)^19^,^20^,^45^. Stitched enhancers and single enhancers were ranked based on H3K27ac signal (merged from two biological replicates) after removing alignments within promoter regions and ENCODE blacklisted regions from the H3K27ac bam file and the corresponding ChIP-seq input (Fig. 2a bottom panel and Supplementary Fig. 6a). We identified 1,067 SEs in MKs (shown in pink in Fig. 2a), made up of 11,860 SE constituents, and 17,790 other enhancers (shown in blue in Fig. 2a), made up of 41,069 IDEAS enhancers (TEs). In EBs we identified 1,287 SEs (shown in pink in Supplementary Fig. 6a), made up of 13,811 constituents, and 17,954 other enhancers (shown in blue in Supplementary Fig. 6a), made up of 41,133 TEs. Overlaps between EB and MK SEs were determined with bedtools v2.22.0 requiring at least 50% of their length to overlap.

### SE opening

We traced the opening of SEs by overlapping SE constituents with MK ATAC-seq or EB DNaseI-seq open chromatin peaks±500 bp. These MK or EB open chromatin peaks were overlapped with ATAC-seq peaks in HSCs, CMPs or MEPs (minimum overlap of 1 bp). CTCF and CD34+ H3K27ac labels were assigned as described above for chromatin opening.

### Differentially acetylated enhancers

To identify differentially acetylated enhancers between MKs and EBs, we used the DiffBind R package (Bioconductor http://bioconductor.org/packages/release/bioc/html/DiffBind.html), using as input the MK and EB enhancer regions identified using IDEAS genome segmentation algorithm and the alignments of H3K27ac and input per cell type (two biological replicates each). The tool collapsed the two sets of enhancers to 68,672 enhancer regions and then counted the number of reads overlapping each region. Sample normalization and differential analysis were then performed using DESeq2 (ref. 46). Figure 1b displays an MA plot for all enhancer regions, highlighting the differential acetylated regions; adjusted *P*-value<0.05 and an absolute log_2_ fold change>1.

### Detection of cell type-specific promoter-interacting regions

The differentially interacting fragments between MKs and EBs were identified using the DESeq2 R package (Bioconductor, https://bioconductor.org/packages/release/bioc/html/DESeq2.html). Interactions with a normalized CHiCAGO score of at least 5 in at least one of the two cell types were tested with standard parameters.

### Region annotation based on PCHi-C

All *Hind*III fragments captured in the PCHi-C (baits) were annotated with the genes whose transcriptional start sites they overlapped (Ensembl v70). Enhancers, SEs and open chromatin peaks were assigned to the genes they interact with using PCHi-C data of the corresponding cell type^16^ by overlapping the region of interest with all possible *Hind*III fragments of the human genome. Regions of interest overlapping prey *Hind*III fragments were assigned to an interacting gene if an interacting bait fragment contained the promoter region of that gene. Interactions were also considered between two bait *Hind*III fragments. Interactions between a bait fragment containing the region of interest and a prey fragment were not considered. For baits that contain transcriptional start sites for more than one gene, all overlapping genes were used to define the interacting gene. If the region of interest overlapped with more than one *Hind*III fragment and/or interacted with more than one bait, interactions of all overlapping fragments and all interacting baits were used. A total of 674 GWAS sentinel SNPs for mean platelet volume, platelet count, platelet distribution width and plateletcrit from Astle *et al*.^1^, were assigned to the gene(s) they most probably influence in a multi-step process (Supplementary Fig. 8a):

1. Based on the VeP prediction^47^, exonic and splice site variants were assigned to the corresponding gene.
2. Variants overlapping exons of genes that were not expressed in our RNA-seq data (FPKM<1) and non-coding variants were overlapped with MK promoters±1 kb that overlap an annotated transcriptional start site (as obtained from the genome segmentation) and assigned to the corresponding gene(s).
3. If an exonic GWAS sentinel SNP was in an element labelled as an enhancer in the IDEAS genome segmentation or if the gene was not expressed in our RNA-seq data (FPKM<1), and the SNP did not overlap a promoter, the variant was assigned to the gene and additionally to the gene(s) of the interacting PCHi-C bait(s).
4. Intronic and intergenic variants were overlapped with *Hind*III fragments and assigned to the genes of the baits interacting with the overlapping fragment.

If there was no interacting bait, we obtained all variants in LD (*r*^2^=1) from the NIHR BioResource—Rare Diseases whole genome sequencing and whole exome sequencing study (https:/bioresource.nihr.ac.uk/rare-diseases/welcome/) of 6,687 subjects, repeated our annotation steps with this set of variants and used their annotations as the sentinel SNP annotation.

We repeated these steps for unassigned variants identifying variants at *r*^2^≥0.9 in the first instance and subsequently at *r*^2^≥0.8. Variants that could not be assigned by LD, either because they had no LD variants or because the LD variants could not be assigned, were assessed for overlap with PCHi-C baits±10 kb and assigned to the gene(s) on the overlapping bait as we know that we lack sensitivity to detect short-range interactions between promoters and regulatory elements^16^.

### GO term enrichment

FIDEA was used to determine enrichment of GO terms in gene lists^48^.

### Protein–protein interaction network

The proteins encoded by the 781 protein-coding genes assigned to a GWAS variant based on PCHi-C and LD data were used as primary baits to develop the protein–protein interaction network and the corresponding UNIPROT protein identifier was obtained. To develop a system level network centered on the core proteins, we initially searched for first-order interactors of the 781 core proteins in public databases. Two different types of resources were used for this initial effort, Reactome^49^ (www.reactome.org) and IntAct^50^ (http://www.ebi.ac.uk/intact/) databases. Network visualization was done using Cytoscape^51^ (http://www.cytoscape.org/).

### CBC-P GWAS hit circular permutation enrichment in regulatory regions

The significance of enrichment of strongly associated GWAS variants in SE was estimated by the circular permutation method. The number of variants significantly associated with platelet traits and residing within SEs was determined. Then *P*-values for all variants in the GWAS study were shifted forward by a random number of variant positions (when an end of a chromosome was reached *P*-values were moved to next chromosome; chromosome one was assumed to follow chromosome 22). The *P*-values were thus shifted 999,999 times and on each occasion SEs were overlaid with significant associations (altered *P*-values were considered when locating strong associations after a shift). *P*-values measuring how likely it is to see at least the number of observed variants within SEs were obtained for both original and shifted data sets. The latter *P*-values were ranked and the rank of the original data set was determined; this rank was divided by 1,000,000 and was reported as an empirical *P*-value. Within each enrichment, the number of platelet variants in SEs was contrasted with the amount of red cell variants residing within the same type of SEs. SEs of another cell type were used to model the background distribution of significant GWAS variants within enhancers. Thus, an enrichment is always relative to other enhancers and is estimated as an enrichment of platelet trait variants versus red cell variants. The same procedure was carried out for other enhancer types—the foreground and background enhancers were exchanged, whereas the sets of platelet and red cell variants stayed the same. The method of shifting *P*-values preserves correlations between nearby variants and is also well suited for dealing with physical clustering of enhancer regions on genome.

The numbers of various types of variants within diverse enhancer regions are summarised in Supplementary Table 10.

### Data availability

BLUEPRINT ChIP-seq data for MKs and EBs were obtained from EGA data sets EGAD00001002362 and EGAD00001002377, respectively. BLUEPRINT RNA-seq data were obtained from EGA study EGAS00001000327. All additional high-throughput sequencing data used in this manuscript have been deposited in EGA under data set EGAD00001001871.

## Additional information

**How to cite this article:** Petersen, R. *et al*. Platelet function is modified by common sequence variation in megakaryocyte super enhancers. *Nat. Commun.* **8,** 16058 doi: 10.1038/ncomms16058 (2017).

**Publisher’s note:** Springer Nature remains neutral with regard to jurisdictional claims in published maps and institutional affiliations.

## Supplementary Material

Supplementary Data 1

Supplementary Data 2

Supplementary Data 3

Supplementary Data 4

Supplementary Data 5

Supplementary Data 6

Supplementary Data 7

Supplementary Data 8

Supplementary Data 9

Supplementary Data 10

Supplementary Information

Peer Review File
