# Supplementary material for: Platelet function is modified by common sequence variation in megakaryocyte super enhancers
Source: Nat Commun. 2017 Jul 13;8:16058. doi: 10.1038/ncomms16058 (PMC5511350; doi:10.1038/ncomms16058)
Supplement: Supplementary Information [file ncomms16058-s11.pdf]

File name: Supplementary Information
Description: Supplementary Figures, Tables and Methods

File name: Supplementary Data 1
Description: Megakaryocyte and erythroblast open chromatin dynamics

File name: Supplementary Data 2
Description: Promoter capture Hi-C interactions in MKs and EBs

File name: Supplementary Data 3
Description: Gene Ontology enrichment of genes connected to MK or EB open chromatin peaks of different categories (Fig. 1a and Supplementary Fig. 2b).

File name: Supplementary Data 4
Description: DESeq results for differential acetylation of MK and EB enhancers and GO enrichments of their connected genes

File name: Supplementary Data 5
Description: Genes differentially expressed between MKs and EBs

File name: Supplementary Data 6
Description: MK and EB super enhancers and GO enrichments of their connected genes

File name: Supplementary Data 7
Description: MK and EB SE open chromatin dynamics

File name: Supplementary Data 8
Description: Platelet GWAS hit annotation based on promoter capture Hi-C

File name: Supplementary Data 9
Description: Enriched GO categories for genes connected to megakaryocyte super enhancers overlapping a GWAS sentinel variant or proxy

File name: Supplementary Data 10
Description: Cytoscape file of Reactome and IntAct interactions between the proteins that were linked to CBC-P variants.

File name: Peer Review File
Description:

Supplementary Information

Supplementary Figures

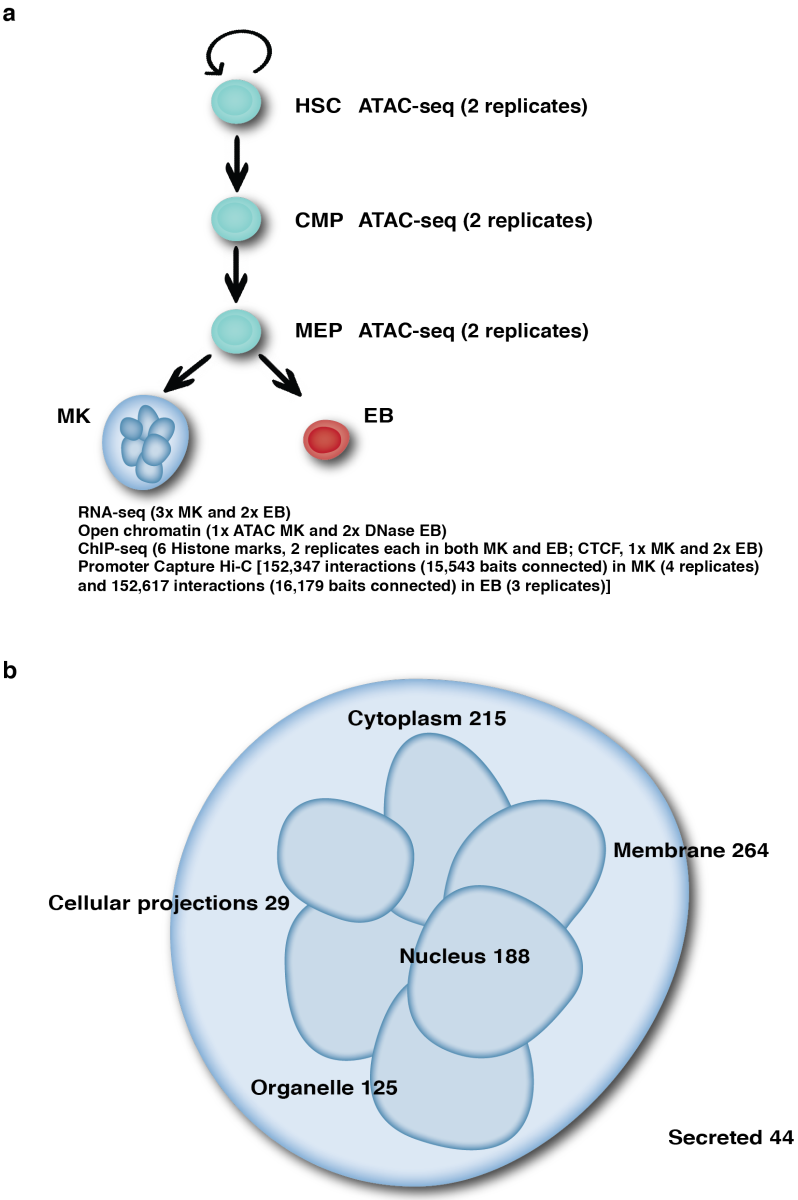

Supplementary Figure 1

Experimental design and cellular localization of genes associated with platelet traits.

a, Segment of the hematopoietic tree with type of assay and replicate number next to each cell type (hematopoietic stem cell, HSC; common myeloid progenitor, CMP; megakaryocyte-erythroid progenitor, MEP; megakaryocyte, MK; erythroblast, EB) used in this study. See Materials and Methods for details of the assays (RNA-seq, ATAC-seq, DNase-seq, ChIP-seq and Promoter Capture Hi-C (PCHi-C)). Populations were defined using the cell surface markers. Hematopoietic stem cells (HSCs): Lin-, CD34+, CD38-, CD90+, CD45RA-. Common myeloid progenitors (CMPs): Lin-, CD34+, CD38+, CD123low, CD45RA-. Megakaryocyte-erythroid progenitors (MEPs): Lin-, CD34+, CD38+, CD123-, CD45RA-. **b**, Cellular localization

of the proteins encoded by the 975 genes connected to the 674 sentinel single nucleotide polymorphisms (SNPs) associated with complete blood count (CBC) measured platelet traits identified by our genome-wide association study (GWAS).

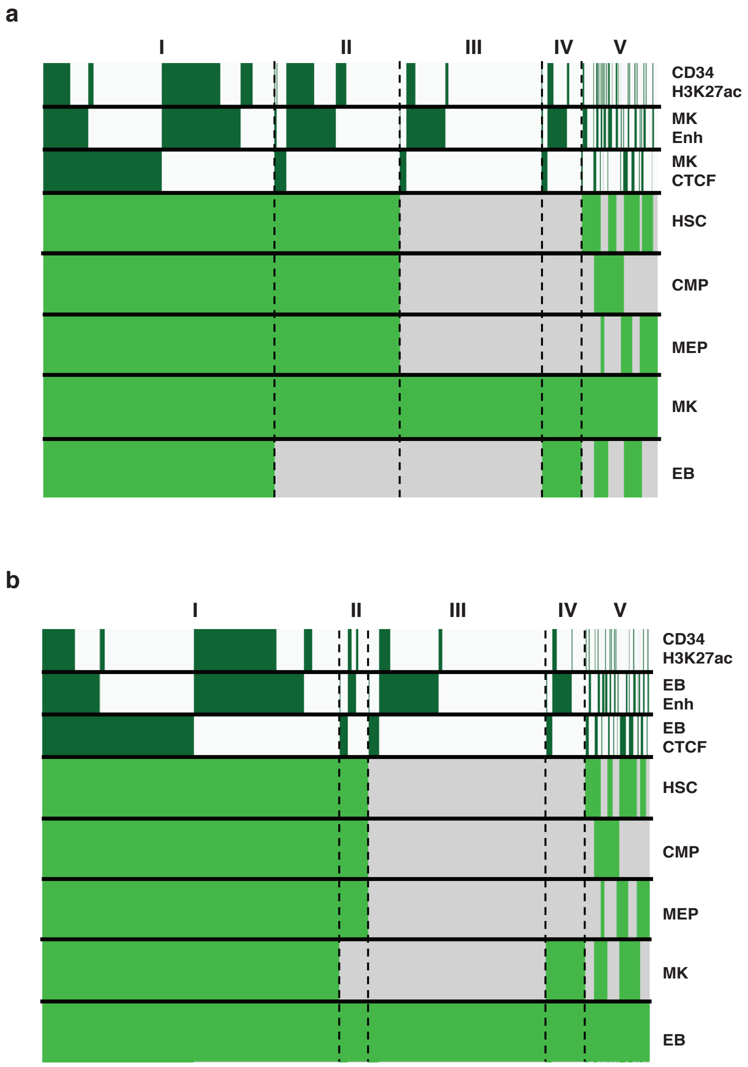

Supplementary Figure 2

Open chromatin dynamics of haematopoiesis.

a, Heatmap depicting MK open chromatin regions (ATAC-seq) dynamics. Bottom five rows show the chromatin status, open (green) or closed (grey), for regions in EBs, MKs, MEPs, CMPs and HSCs (bottom to top). The top three rows show the overlap of MK open chromatin regions with (top to bottom): H3K27ac peaks in CD34+ cells, MK enhancers as identified by genome segmentation and MK CTCF binding sites (dark green, present). The four most prevalent categories are separated by dashed black lines (as in **Fig. 1a**), category V represents all remaining combinations of open chromatin regions. **b**, Heatmap depicting EB open chromatin regions (DNase-seq) dynamics. Bottom five rows show, the chromatin status, open (green) or closed (grey), of each region in EBs, MKs, MEPs, CMPs and HSCs (top

to bottom). The top three rows show the overlap of EB open chromatin regions with (top to bottom): H3K27ac peaks in CD34+ cells, EB enhancers as identified by genome segmentation and EB CTCF peaks.

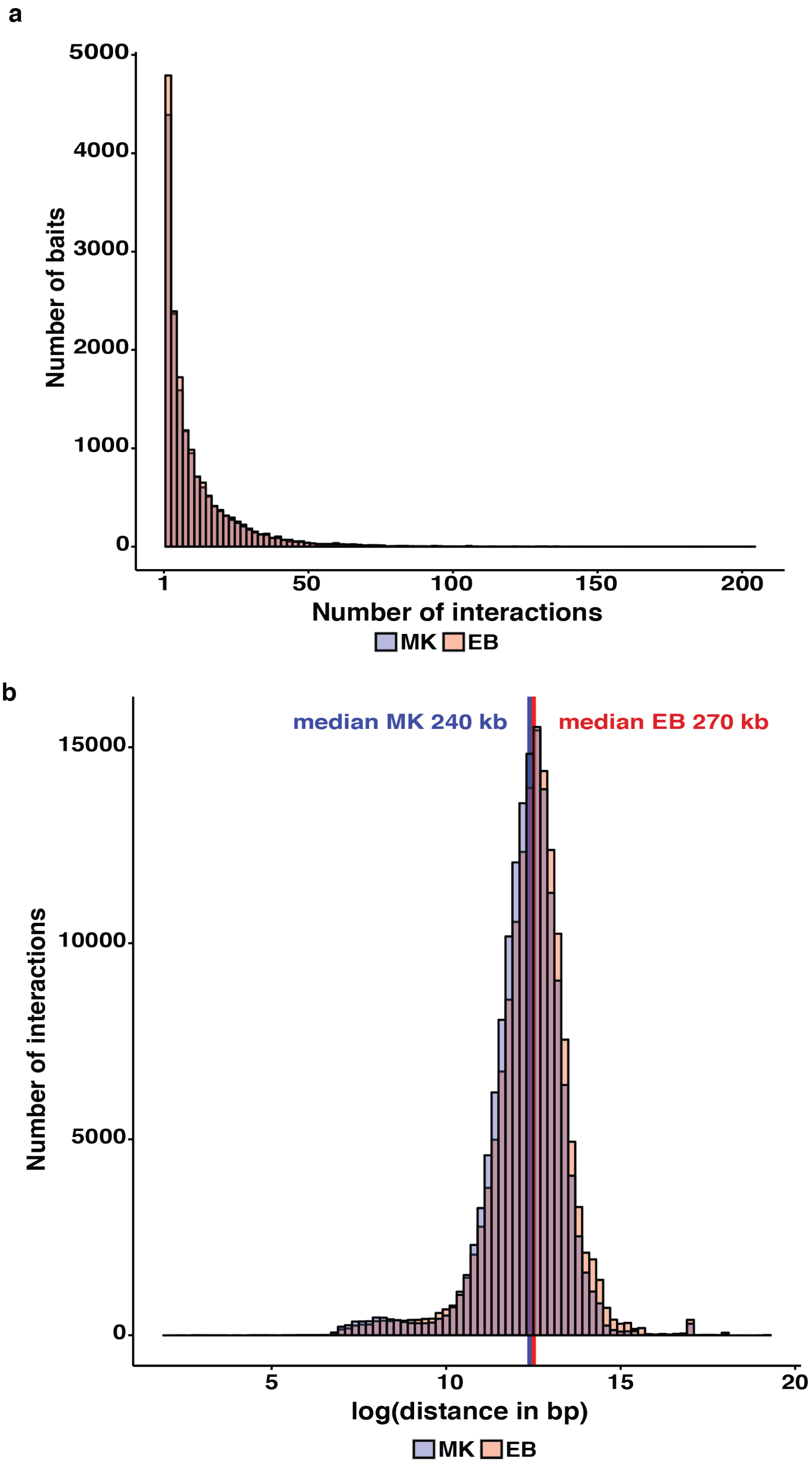

Supplementary Figure 3

Summary of promoter capture Hi-C interactions in megakaryocytes and erythroblasts.

a, Distribution of the number of PCHi-C interactions per bait in MKs (blue) and EBs (red). **b**,

Distribution of distances between interacting fragments in MKs (blue) and EBs (red).

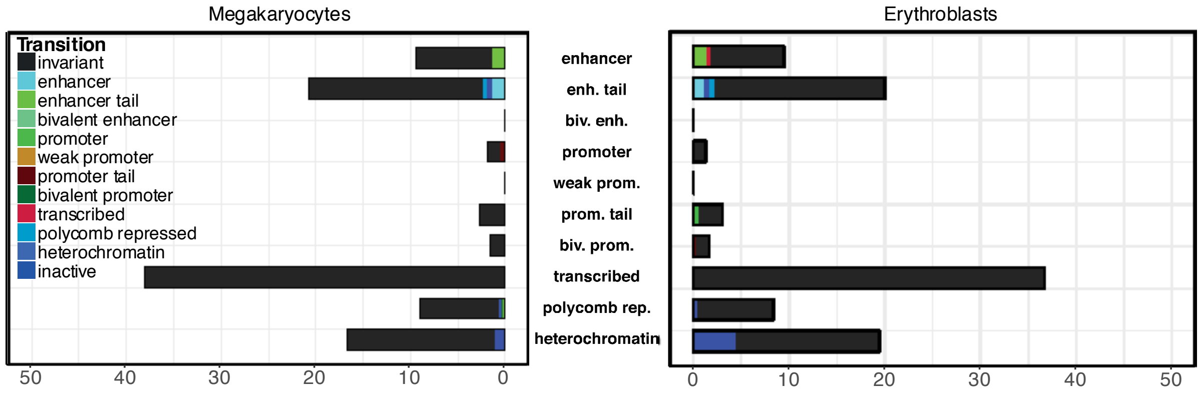

Supplementary Figure 4

Genome segmentation overlap between megakaryocytes and erythroblasts.

Bar plot reporting the percentage of genomic space occupied by each functional state in MKs (left) and EBs (right) as defined by segmentation analysis. Colored sections of bars represent different states in MKs and EBs as indicated in the legend. Black sections represent regions with the same segmentation state in both cell types. The inactive state has been omitted for clarity. enh. tail., enhancer tail; biv. enh., bivalent enhancer; weak prom., weak promoter; prom. tail, promoter tail; biv. prom., bivalent promoter; Polycomb rep., Polycomb repressed.

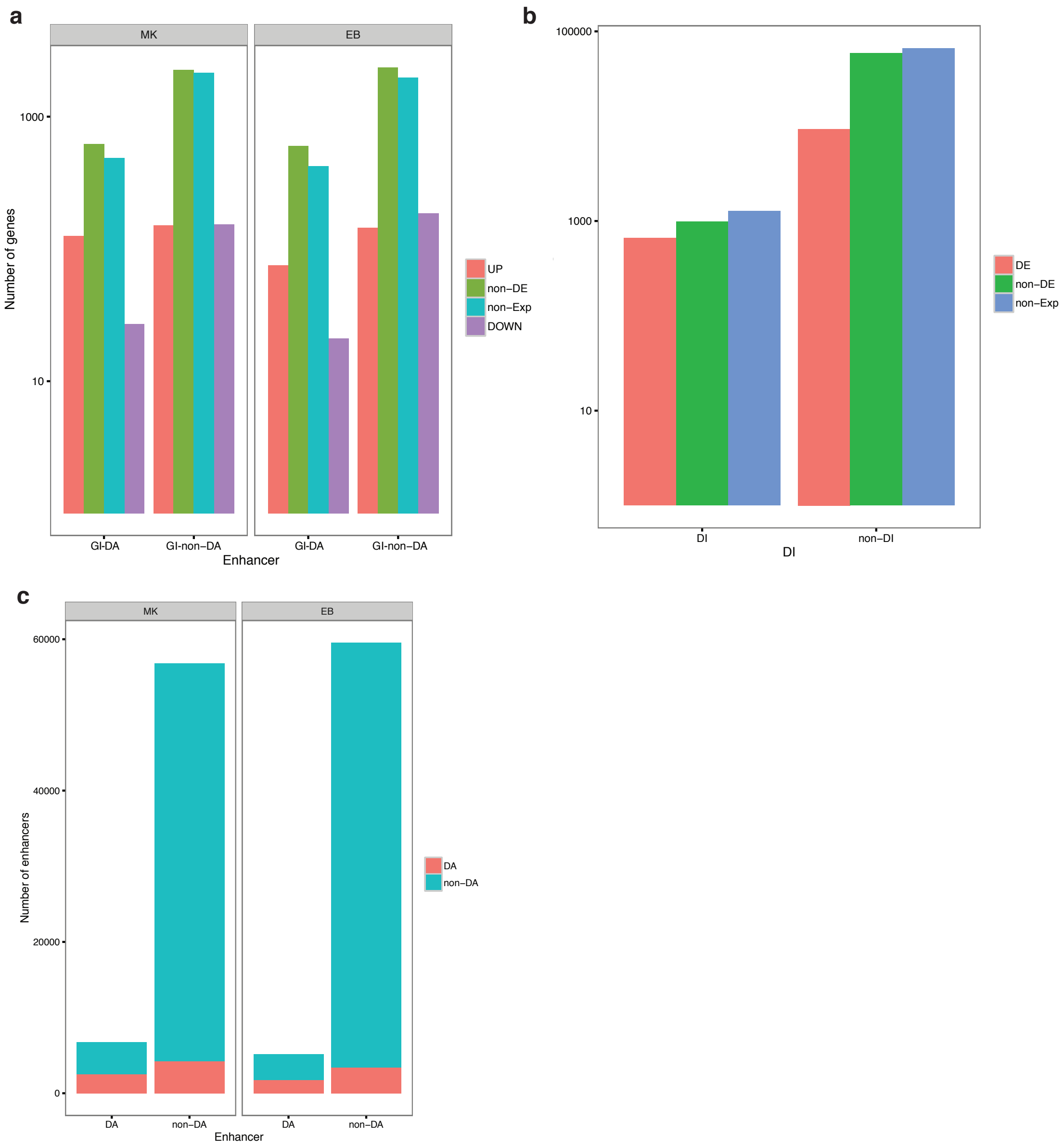

Supplementary Figure 5

Patterns of interactions of open chromatin regions in megakaryocytes and erythroblasts.

a, Acetylated enhancers are associated with differential gene expression. Genes that interact with at least one differential acetylated (DA) enhancer (GI-DA) with increased acetylation in MKs (left) or EBs (right) are enriched for up regulated genes (UP) in MKs and EBs respectively when compared to the cumulative number of non-differentially expressed genes (non-DE), non expressed genes (non-Exp) and down regulated genes (DOWN) (MK Fisher's Exact Test $P < 2.2\text{x}10^{-16}$, odds ratio (OR) 3.3, EB Fisher's Exact Test $P < 1.2\text{x}10^{-07}$, OR 2.2). **b**, Differentially interacting (DI) fragments show enrichment for interactions with promoters of differentially expressed (DE) genes (Fisher's Exact Test $P < 2.2\text{x}10^{-16}$, OR 3.9). Non-differentially expressed genes (non-DE); non- expressed genes (non-Exp). **c**, Differentially acetylated enhancers cluster in the genome. DA are more likely to have a DA neighbour than a non-DA neighbour in both MKs and EBs (Fisher's Exact Test $P < 2.2\text{x}10^{-16}$, OR for MKs 7.3; OR for EBs 8.2).

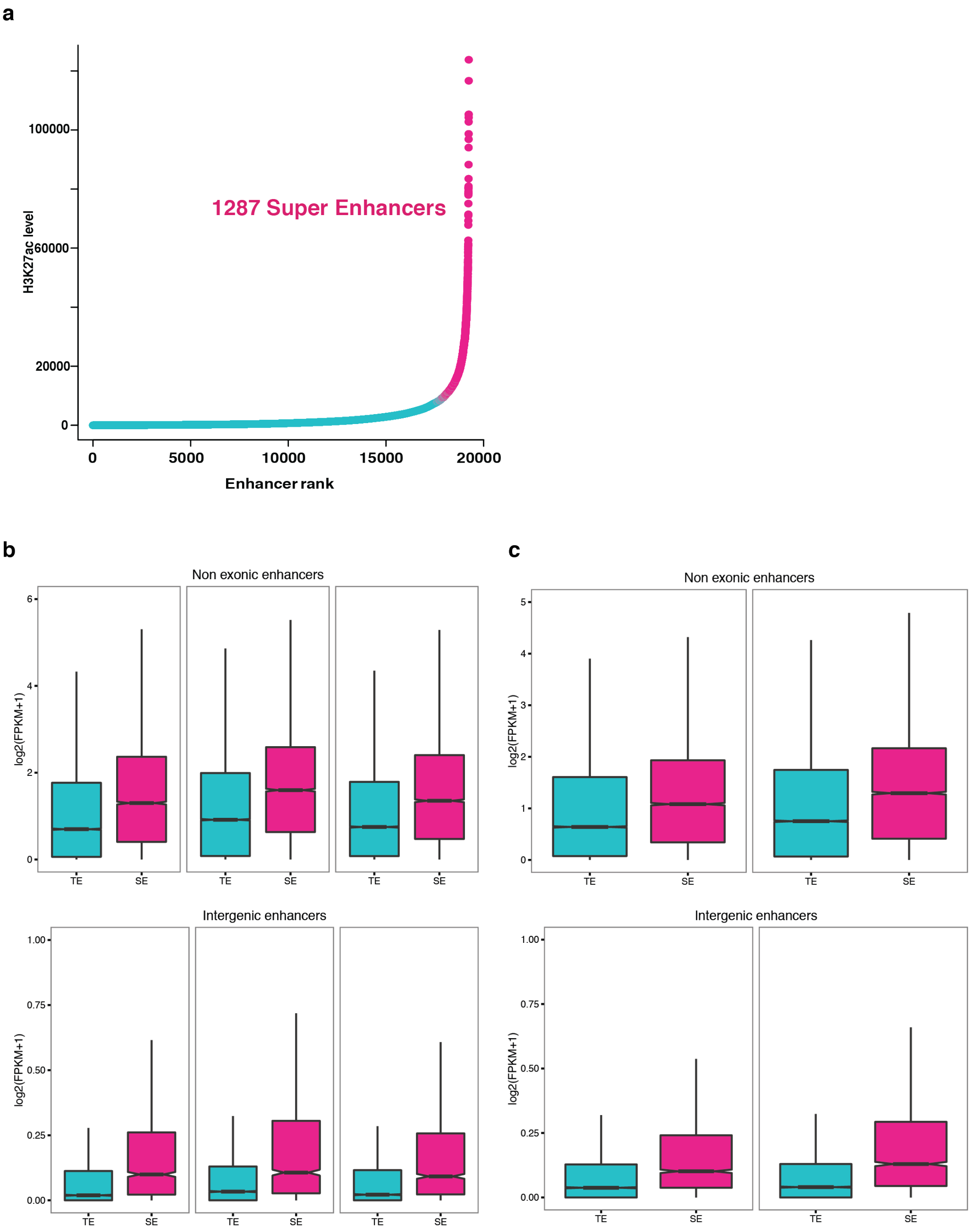

Supplementary Figure 6

Identification of super enhancers in erythroblasts and evidence of transcription at super enhancers in megakaryocytes and erythroblasts.

a, Ranking of enhancers based on H3K27ac signal intensities in EBs to define 1,287 super enhancers (SEs). Stitched enhancer regions with high H3K27ac signal intensities were identified as SEs (pink) with the low H3K27ac signal enhancers defined as other enhancers (blue). Non-exonic and intergenic SEs constituents (pink) have higher transcription than TEs (blue) for **b**, MKs and **c**, EBs. All differences are significant (Wilcoxon test $P < 10^{-10}$). The results of three and two biological replicates are shown for MKs and EBs, respectively (box plot: line indicates median, upper and lower box margins indicate first and third quartile).

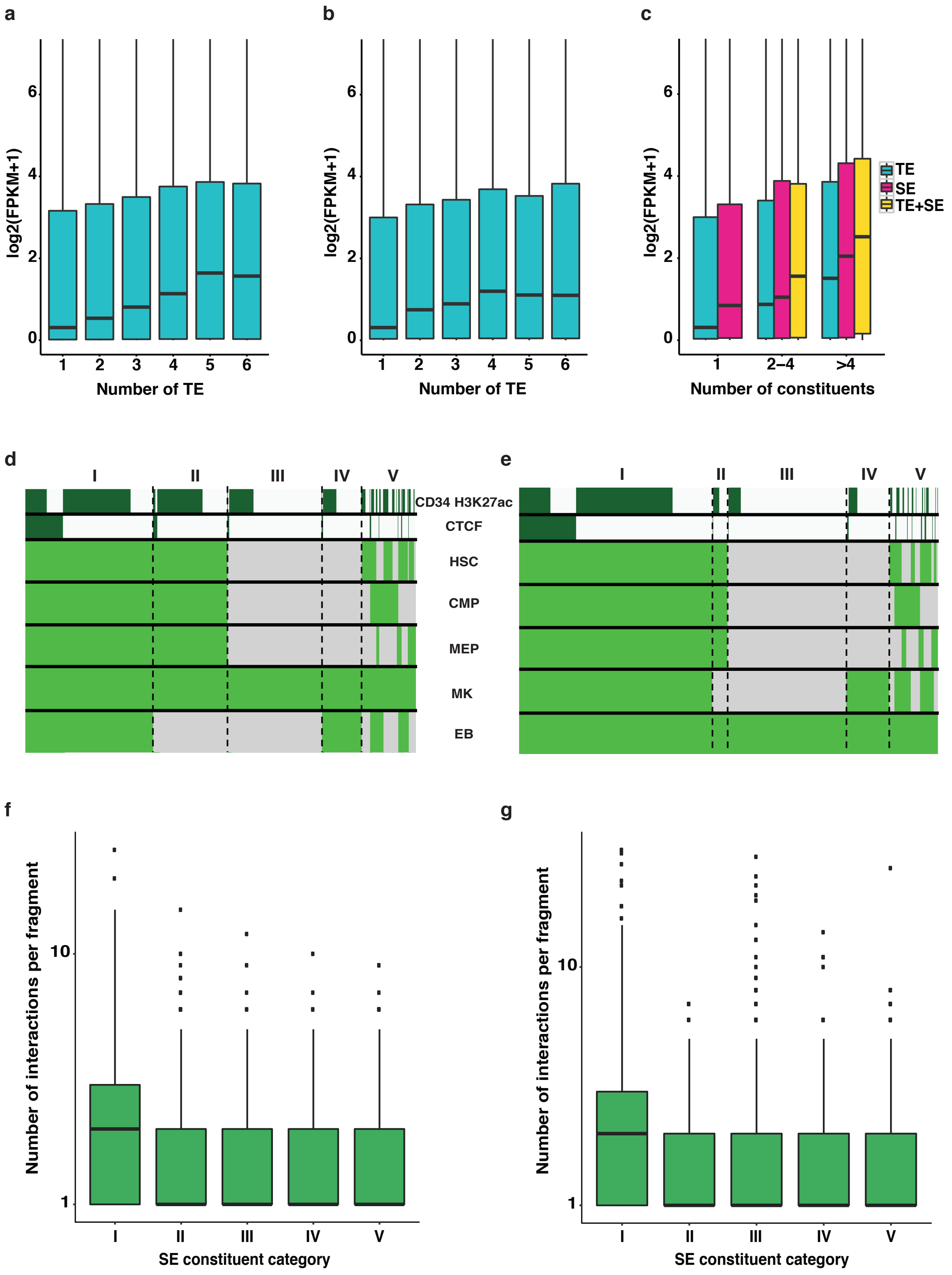

Supplementary Figure 7

Effect of typical and super enhancers on gene expression and opening dynamics.

Gene expression in **a**, MKs and **b**, EBs for genes connected to TEs only, categorized depending on the number of TEs interacting with the gene (*P* values for two-tailed Wilcoxon test in **Supplementary Table 3**; box plot: line indicates median, upper and lower box

margins indicate first and third quartile). **c**, Gene expression in EBs, for genes connected to TE only (blue), SE constituents only (pink), or TE and SE constituents (yellow) (*P* values for two-tailed Wilcoxon test in **Supplementary Table 3**; box plot: line indicates median, upper and lower box margins indicate first and third quartile). Opening dynamics of constituents of **d.** MK SEs and **e**. EB SEs during hematopoietic differentiation. Open chromatin regions overlapping with SE constituents in HSCs, CMPs, MEPs (ATAC-seq) and EBs (DNase-seq). H3K27ac in CD34+ cells and EB CTCF binding sites added for comparison (color legend as in **Fig. 1a and categories as in Supplementary Fig. 2a**). **f** and **g** Fragments in SE class I have significantly higher interactions than in other SE classes in MKs, Wilcoxon test *P* values MK: I-II $6.2x10^{-7}$; I-II $7.5x10^{-11}$, I-IV 6.5 $x10^{-5}$, I-V 4.5 $x10^{-5}$ and in EB (**g**), Wilcoxon test *P* values EB: I-II $6.1x10^{-4}$; I-II $8x10^{-20}$, I-IV 7.2 $x10^{-10}$, I-V 4.5 $x10^{-7}$.

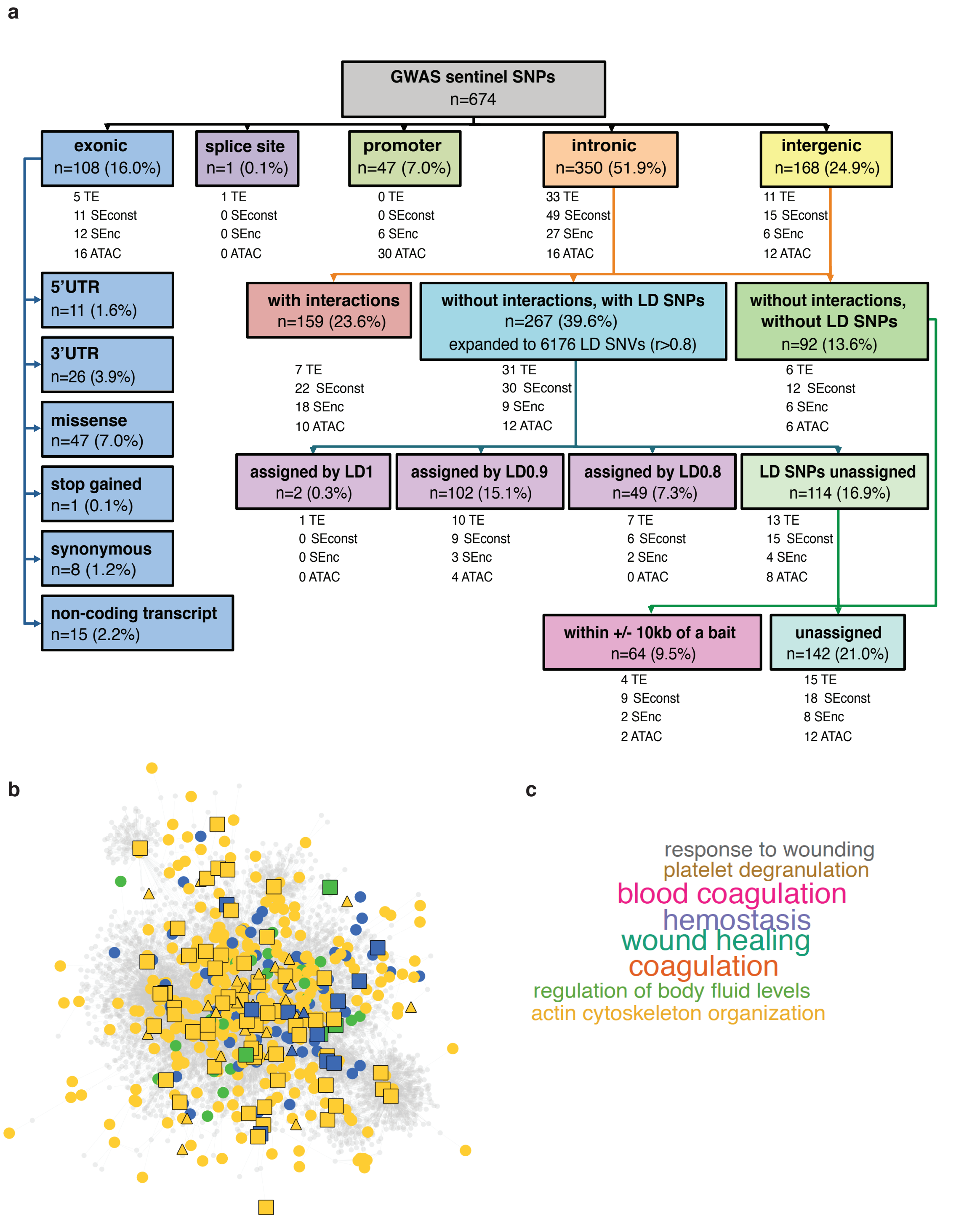

Supplementary Figure 8

Annotation and protein-protein interaction network of genome-wide association study single nucleotide variants.

a, Of the 674 unique GWAS sentinel variants associated with CBC-measured platelet traits (CBC-P)[27], 532 were assigned to the gene(s) they most likely regulate by prioritizing their location, functional annotation and long-range interactions in MKs. Of these, 153 sentinel variants were assigned to genes by using single nucleotide polymorphisms (SNPs) in linkage disequilibrium (LD) at $r^2 > 0.8$ (**Fig. 3a**, Materials and Methods). Many sentinel variants (37%)

were located in typical enhancers (TEs), super enhancer constituents (SEconst), super enhancers outside of constituents (SEnc) or ATAC-seq peaks (ATAC). **b**, Protein-protein interaction network of 4,235 nodes (proteins) and 18,550 edges. The network was generated by deriving the interactions of 781 protein coding genes (a subset if 975 genes assigned to GWAS sentinel variants [**Fig. 3b**]; the remaining genes are non-protein coding) from the Reactome and IntAct databases. Nodes representing 526 proteins are colored according to the location of the associated sentinel variant (as per **Fig. 3a**): exonic (blue), MK promoter (green) and intronic or intergenic (yellow) and the shapes indicate the type of elements the variants are located in: TEs (triangles; 30), SEs (squares; 72) or element lacking an enhancer signature (circles; 424). A digital version of the network with attributes can be downloaded as **Supplementary Data 10**. Information about the attributes can be found in Materials and Methods. **c**, Word cloud of the GO enrichment for Biological Process of all protein-coding genes linked to MK SEs that harbour a CBC-P sentinel SNP or proxy.

Supplementary Tables

Supplementary Table 1 Summary of long-distance interactions detected by promoter capture Hi-C in erythroblasts and megakaryocytes

	**MK**	**EB**
**Total interactions**		
number of total interactions	152347	152617
number of hind3 fragments involved	98159	90184
number of baits involved	15543	16179
number of preys involved	82616	74005
median number of interactions per bait (all baits)	3	3
median number of interactions per bait ("active" baits)	6	5
size of genome covered in bp	381634638	362478856
median distance of interacting fragments in bp (for intrachromosomal interactions)	239866	270835
**Promoter-promoter interactions**		
number of promoter-promoter interactions	14209	18529
number of baits involved	10971	12296
median number of interactions per bait ("active" baits)	2	2
size of genome covered by promoter-promoter interactions in bp	85210604	99813681
median distance of interacting baits (for intrachromosomal interactions)	214731	255304

Supplementary Table 2 Tests and *P* values for enrichment of CTCF and open chromatin region in PCHi-C interacting fragments

	Model	Beta	*P* value
CTCF peak count in HindIII-fragments versus HindIII fragment is interacting (binary) + fragment length	Zero-inflated negative binomial model	1.730239	<2 $x10^{-16}$
Open chromatin peak count in HindIII-fragments versus HindIII fragment is interacting (binary) + fragment length	Zero-inflated negative binomial model	1.412549	<2 $x10^{-16}$
CTCF peak count that lie in open chromatin peaks versus overlapping HindIII fragment is interacting (binary) + fragment length	Negative binomial model	1.849302	<2 $x10^{-16}$
CTCF peak count that lie outside open chromatin peaks versus HindIII fragment is interacting (binary) + fragment length	Negative binomial model	0.8137318	<2 $x10^{-16}$

Supplementary Table 3 *P* values for two-tailed Wilcoxon test of gene expression

Condition1	Condition2	*P* value MKs	*P* value EBs
1 TE	1 SE	9.82×10^{-3}	1.73×10^{-5}
2-4 TE	2-4 SE	6.31×10^{-3}	2.42×10^{-2}
2-4 TE	2-4 TE+SE	9.02×10^{-4}	1.47×10^{-6}
2-4 SE	2-4 TE+SE	9.15×10^{-1}	2.02×10^{-1}
>4 TE	>4 SE	7.54×10^{-1}	5.19×10^{-2}
>4 TE	>4 TE+SE	8.45×10^{-7}	1.15×10^{-14}
>4 SE	>4 TE+SE	7.37×10^{-2}	1.18×10^{-1}
1 TE	2-4 TE	2.89×10^{-8}	2.43×10^{-12}
2-4 TE	>4 TE	1.74×10^{-11}	4.29×10^{-8}
1 TE	>4 TE	2.20×10^{-16}	2.20×10^{-16}
1 SE	2-4 SE	1.38×10^{-1}	3.89×10^{-1}
2-4 SE	>4 SE	5.34×10^{-1}	3.08×10^{-2}
1 SE	>4 SE	8.16×10^{-2}	2.94×10^{-3}
2-4 TE+SE	>4 TE+SE	1.15×10^{-4}	5.30×10^{-7}
1 TE	2 TE	4.14×10^{-3}	1.52×10^{-5}
2 TE	3 TE	2.84×10^{-2}	1.02×10^{-1}
3 TE	4 TE	1.42×10^{-1}	1.27×10^{-1}

4 TE	5 TE	2.79×10^{-1}	8.67×10^{-1}
5 TE	6 TE	7.96×10^{-1}	8.19×10^{-1}
1TE	3TE	1.83×10^{-6}	3.21×10^{-8}
2TE	4TE	7.72×10^{-4}	3.16×10^{-3}
3TE	5TE	1.08×10^{-2}	2.46×10^{-1}
4TE	6TE	4.74×10^{-1}	8.57×10^{-1}

The significance of gene expression differences between genes connected to different numbers of TE, SE constituents or TE and SE constituents as shown in **Fig. 2c** and **Supplementary Fig. 7a-c** was tested.

Supplementary Table 4: *P* values circular permutation analysis

Cell type of reference enhancer	Type of reference enhancer	Empirical *P* value of enrichment
EB unique	super	1.00E-06
EB unique	low intensity	1.99E-02
shared	super	1.00E-05
shared	low intensity	5.46E-01
EB unique	super	8.80E-05
EB unique	low intensity	2.16E-04
MK unique	low intensity	2.90E-03
shared	low intensity	9.16E-01
EB unique	low intensity	4.48E-01

All *P* values are based on a permutation test involving 999,999 simulated data sets of locations of significantly associated GWAS variants. The first two columns describe the enhancer for which an enrichment of variants is being assessed. The third and the fourth columns identify the reference enhancer against which the relative enrichment in platelet variants versus red cell variants was estimated. The first six rows describe comparisons of enhancers belonging to different cell types. The last three rows contrast super and low intensity enhancers for the same cell type.

Supplementary Table 5: Univariate linear regression of clotting phenotypes of whole blood flowed over different prothrombotic surfaces on allelic scores at four single nucleotide variants

	**rs10886430**	**rs3557**	**rs1613662**	**rs2363877**
Collagen I,Itg	0.533040681	0.04744176	0.166661628	0.690612086
Collagen I,MoSc	0.213111445	0.684126739	0.106554668	0.127292912
Collagen I,PltSac	0.809026493	0.106668824	0.004961335	0.05239416
Collagen I,PSel	0.360307566	0.259959728	0.349683002	0.591777205
Collagen I,PSSac	0.01980555	0.644662246	0.698630164	0.239849111

Collagen I,ThContrSc	0.482100595	0.157832326	0.933753143	0.704035092
Collagen I,ThMultilayerSc	0.517868253	0.442880016	0.910169353	0.826090583
Collagen I,ThSac	0.151579446	0.93048203	0.555885609	0.787838856
Collagen III,Itg	0.072142263	0.003390593	0.896677351	0.248414986
Collagen III,MoSc	0.540767347	0.888802847	0.363004619	0.804963633
Collagen III,PltSac	0.304947087	0.242801513	0.018492137	0.886437916
Collagen III,PSel	0.484208136	0.557246519	0.200806592	0.367746033
Collagen III,PSSac	0.726966012	0.799885861	0.201530062	0.731538227
Collagen III,ThContrSc	0.460426406	0.53017856	0.276300626	0.649540872
Collagen III,ThMultilayerSc	0.458155392	0.809717218	0.240726165	0.511008668
Collagen III,ThSac	0.370226937	0.53328165	0.144929275	0.384977673
VWF laminin,Itg	0.466572383	0.071584092	0.943855842	0.413276893
VWF laminin,MoSc	0.77904711	0.604755522	0.898870759	0.38617003
VWF laminin,PltSac	0.148133394	0.884637164	0.190101745	0.958875077
VWF laminin,PSel	0.339780433	0.407043237	0.367540675	0.615001254
VWF laminin,PSSac	0.417364427	0.200855112	0.374926156	0.972335336
VWF laminin,ThContrSc	0.72060647	0.468879861	0.364753101	0.544357937
VWF laminin,ThMultilayerSc	0.737847376	0.406792238	0.7827218	0.363210602
VWF laminin,ThSac	0.710106621	0.796591588	0.431692615	0.810643738
VWF-BP GFOGER-GPO,Itg	0.348900492	0.014129478	0.396310676	0.682088685
VWF-BP GFOGER-GPO,MoSc	0.502128279	0.948324958	0.473712682	0.735995011
VWF-BP GFOGER-GPO,PltSac	0.909700277	0.91442661	0.247425107	0.858998779
VWF-BP GFOGER-GPO,PSel	0.346314029	0.343373334	0.353405739	0.765024452
VWF-BP GFOGER-GPO,PSSac	0.388665952	0.718438036	0.574739522	0.700426627
VWF-BP GFOGER-GPO,ThContrSc	0.621600919	0.73895224	0.750753134	0.891190905
VWF-BP GFOGER-GPO,ThMultilayerSc	0.361849012	0.727532902	0.932126135	0.636258366
VWF-BP GFOGER-GPO,ThSac	0.623529282	0.521485079	0.7402795	0.98279583
VWF-BP rhyodocytin,Itg	0.414512674	0.525480179	0.740157694	0.357001065
VWF-BP rhyodocytin,MoSc	0.592105503	0.095979382	0.253351039	0.942556407
VWF-BP rhyodocytin,PltSac	0.29697824	0.025200439	0.266532616	0.105789324
VWF-BP rhyodocytin,PSel	0.210835278	0.320417699	0.504229896	0.04801866
VWF-BP rhyodocytin,PSSac	0.238899185	0.191552342	0.460999529	0.708608075
VWF-BP rhyodocytin,ThContrSc	0.270925461	0.075366478	0.033850991	0.529420241
VWF-BP rhyodocytin,ThMultilayerSc	0.245963871	0.109520245	0.039308066	0.475134068
VWF-BP rhyodocytin,ThSac	0.098428681	0.089146567	0.211765218	0.353047494

The measurements for each clotting phenotype and prothrombotic surface were adjusted for sex by linear regression and quantile normalized. Association of each phenotype with allelic score for each single nucleotide variant was then assessed using a likelihood ratio test under a linear regression model.

Supplementary Table 6: ChIP Antibodies

Target	Company	Catalogue / Lot No.	Amount in

			µg/ChIP/million cells
H3K4me1	Diagenode	pAB-194-050 (C15410194)/ A1863-001D	0.5
H3K4me3	Diagenode	pAb-003-050/C1541003/ A5051-001P	1
H3K9me3	Diagenode	pAB-193-050/C15410193/A1671-001P	2
H3K27me3	Diagenode	pAb-195-050/C15410195/ A1811-001P	1
H3K27ac	Diagenode	pAb-196-050 (C15410196)/ A1723-0041D	1
H3K36me3	Diagenode	pAB-192-050/A1847-001P	0.5
CTCF	Diagenode	K08121004/ iTF002	0.25

Supplementary Table 7: CRISPR-Cas9 sgRNAs

Name	Target sequence	PAM (NGG)	Forward Oligonucleotide	Reverse Oligonucleotide
sgRNA1	ATACTCGGAGAGTGACG GGGAGG	AGG	caccgTACTCGGAGAGTG ACGGGG	aaacCCCCGTCACTCTCCG AGTAC
sgRNA2	CTTCCAGAGGAGACTTG CAGGGG	GGG	gaccgTTCCAGAGGAGAC TTGCAG	aaacCTGCAAGTCTCCTCT GGAAC

Supplementary Table 8: Super enhancer genotyping oligonucleotides

Non-targeted cells	GAGGTGTGGAGGGAGTGG	ATGGTCTCTGTGGGCTTAGG	971bp
Non-targeted cells	GAGCAGAGAGAAGGGAAGCA	CCCCAACAGCCAGCTTTTG	698bp

Supplementary Table 9: Oligonucleotides used for VWF, CD9 and GUSB qRT-PCR (all exon spanning)

VWF_Exon20/21_F	CGACGGGCTCAAATACCTGT
VWF_Exon20/21_R	GGGTGGCTGCATCCCTTATT
CD9_Exon5/6/6_F	TCCACTATGCGTTGAACTGCT
CD9_Exon5/6/6_R	TCACGGTGAAGGTTTCGAGT
GUSB_Exon11/12_F	ACGTGGTTGGAGAGCTCATT
GUSB_Exon11/12_R	CTCTGCCGAGTGAAGATCCC

Supplementary Table 10: Number of GWAS variants in enhancers

	% of genome covered	Total sentinel variants in enhancer	Platelet variants in enhancer	Red cell variants in enhancer
MK unique SE	0.20	33	19	4
Shared SE	0.82	171	46	72
EB unique SE	0.25	53	6	36
MK unique other enhancer	0.23	17	5	6
Shared other enhancer	0.99	94	25	21

EB unique other enhancer	0.29	31	4	18

List of Supplementary Data

Supplementary Data 1 Megakaryocyte and erythroblast open chromatin dynamics
Numbers of megakaryocyte (MK) ATAC-seq peaks (sheet "MK") and erythroblast (EB) DNase-seq peaks (sheet "EB") shown in the different categories of **Fig. 1a** and **Supplementary Fig. 2**, respectively.

Supplementary Data 2 Promoter capture Hi-C interactions in MKs and EBs
Data originate from Javierre et al.

Supplementary Data 3 Gene Ontology enrichment of genes connected to MK or EB open chromatin peaks of different categories (Fig. 1a and Supplementary Fig. 2b)
The name of the spreadsheet indicates the cell type and peak category.

Supplementary Data 4 DESeq results for differential acetylation of MK and EB enhancers and GO enrichments of their connected genes
Column "enhancer.shared" indicates whether the enhancer overlaps an enhancer in the second cell type (1) or not (0). Significantly enriched (adjusted *P* value < 0.05) gene ontology (GO) categories for "biological process" of the genes linked to differentially acetylated enhancers (shown in blue and red in **Fig. 1b** for MKs and EBs, respectively) are listed.

Supplementary Data 5 Genes differentially expressed between MKs and EBs
MMSEQ quantification followed by MMDIFF differential expression analysis carried out on RNA-seq data identified 1546 genes as significantly differentially expressed between MK and EB (posterior probability > 0.5). Mean FPKM obtained from two biological replicates for EB and three biological replicates for MK, log fold change and the posterior probability of differential expression are shown.

Supplementary Data 6 MK and EB super enhancers and GO enrichments of their connected genes
ID, position and annotation of 1,067 SEs identified in MKs (**Fig. 2a**, sheet "MK_SE") and 1,287 SEs identified in EBs (**Supplementary Fig. 6a**, sheet "EB_SE"). The number of enhancer constituents stitched to obtain this SE (NUM_LOCI), their size (CONSTITUENT_SIZE) as well as the normalized H3K27ac signal, input signal and enhancer rank as obtained by ROSE are given together with the size of the SE. Columns "hind3_fragment", "overlapping_gene_hgnc" and "overlapping_gene_ensembl_gene_id" describe the PCHi-C *Hind*III fragment overlapped by the SE. Columns "interacting_hind3_fragment", "interacting_genes_hgnc" and "interacting_esembl_gene_id" describe the PCHi-C bait

fragments to which the SE is connected. FIDEA results for all significantly enriched (adjusted *P* value < 0.05) GO categories for “biological process” of the genes linked to SEs are shown in spread sheets “MK_SE_GO” and “EB_SE_GO”.

Supplementary Data 7 MK and EB SE open chromatin dynamics
Numbers of MK ATAC-seq peaks overlapping SE constituents shown in the different categories of **Fig. 2d** and Supplementary **Fig. 7d** as well as numbers of EB DNase-seq peaks overlapping SE constituents shown in the different categories of **Supplementary Fig. 7e** are indicated.

Supplementary Data 8 Platelet GWAS hit annotation based on promoter capture Hi-C
Clumped GWAS sentinel SNVs for the four platelet traits platelet count (#PLT), mean platelet volume (MPV), plateletcrit (PCT) and platelet distribution width (PDW) (CBC-P) along with their VEP annotations were obtained from Astle *et*. al[1] and assigned to the gene(s) they most likely influence in a multi-step process explained in Material and Methods and depicted in **Supplementary Fig. 8a**. Overlaps with MK enhancers (E_status), ATAC-seq peaks and CTCF peaks, overlapping *Hind*III fragments and their annotation (columns AI-AK) and interacting *Hind*III baits and their annotation (columns AL-AN), the expression of the VEP-assigned gene, overlap with genomic features, the gene whose annotated transcription start site overlaps the promoter (for variants located in promoter regions), the genes the variant was assigned to (“genes_assigned”) and the strategy for the assignment (“assigned_via”) are reported.

Supplementary Data 9 Enriched GO categories for genes connected to megakaryocyte super enhancers overlapping a GWAS sentinel variant or proxy
Significantly enriched (adjusted *P* value < 0.05) GO categories for “biological process” of the 290 genes linked to MK SE harboring a CBC-P variant or proxy.

Supplementary Data 10 Cytoscape file of Reactome and IntAct interactions between the proteins that were linked to CBC-P variants.

Supplementary Methods

Multimodular platelet activation in thrombus formation
Blood samples (500µL) were recalcified with 7.5mM $CaCl_2$ and 3.75mM $MgCl_2$ in the presence of 40µM Phe-Pro-Arg dichloro methylketone, 5U/ml heparin and 50U/ml fragmin. Samples were perfused through a 50mM deep parallel-plate perfusion chamber (Maastricht chamber), for 4 minutes at a wall shear rate of 1000$seconds^{-1}$ over micro spotted surfaces containing (adhesive platelet receptors involved underlined): collagen type I (α2β1, GPVI, GPIb); and VWF-BP/rhodocytin (BioSource; GPIb, CLEC2). Representative brightfield images (2/microspot) were captured with an EVOS-FL microscope (Thermo Fisher Scientific),

equipped with an Olympus UPLSAPO 60x oil immersion objective, immediately after blood flow, during perfusion at 1000seconds^{-1} with labeling buffer (10mM HEPES pH 7.45, 136mM NaCl, 2.7mM KCl, 2mM $MgCl_2$, 1mM $CaCl_2$, 5mg/ml glucose, 1mg/ml bovine serum albumin, 1U/ml heparin, 1:100 FITC anti-fibrinogen Mab, DAKO, and 80μg/ml Alexa Fluor 647-anti-CD62P mAb, Biolegend). After 1.5 minutes, labeling was completed by 2 minutes of stasis, after which residual label was removed by post-perfusion with label-free HEPES buffer. Representative fluorescence images (3/microspot) were recorded quasi-simultaneously at 470±22, and 628±40nm excitation. Recorded brightfield and fluorescence images were analyzed in duplicate for thrombus size, fibrinogen binding and PSel expression (surface area coverage in %) by observers blinded to the sample genotype, using semi-automated scripts written in Fiji (ImageJ, http://imagej.net/), requiring manual threshold setting. Parameter data from multiple images/spot were averaged; mean results from duplicate runs per subject were averaged, unless artifacts were observed (air bubbles, fibrin clots). Data from 93 subjects were adjusted for sex by linear regression within each agonist and phenotype combination and quantile normalized. Marginal association between the genotype at a SNP and the sex-adjusted, quantile normalized data was assessed by linear regression.

Genotype-Phenotype subjects

For platelet function experiments and CD9 phenotyping, blood samples were obtained from whole blood or apheresis donors from the NHS Blood and Transplant blood donor clinic in Cambridge after informed consent (Association between sequence polymorphisms and functional variation in blood cells and their precursors in a large cohort of donors, REC ref 05/Q0104/27, Cambridge East Research Ethics Committee). All other phenotyping was performed on blood samples from NIHR Cambridge BioResource donors (blood group O only) after informed consent (Genetic analysis of platelets in healthy individuals, REC ref 10/H0304/65, Cambridge East Research Ethics Committee). For both studies blood was drawn from the antecubital fossa, via a 21-gauge butterfly needle and a Vacutainer using a standardized protocol. The first 3ml of blood were discarded to avoid the risk of platelet activation. Subsequent samples were taken into 3.2% trisodium citrate.

VWF quantification in platelet lysates and plasma

Platelet rich plasma (PRP) was generated from citrated blood by centrifugation at 150xg, room temperature, 3 times, 20 minutes each. PRP (4ml) was centrifuged at 1000xg to separate the plasma supernatant and platelet pellet. The platelet pellet was washed twice in PBS/EDTA (1000xg) and resuspended in 500μL of lysis buffer29. Polyclonal antibody against human von Willebrand Factor (VWF) (Dako, A0082 at 10μg/ml) was bound to a 96-well enzyme-linked immunosorbent assay (ELISA) plate and incubated at 4°C overnight. Plasma (1/100 and 1/300) or platelet lysate (1/50 and 1/100) was added and incubated at room temperature for 2 hours. An HRP-conjugated polyclonal antibody against human VWF (Dako, P0226 at 1/3000) was used to detect the amount of VWF bound to the primary antibody. Optical density was measured on an ELISA reader (450nm). A dilution series of human VWF (0 - 10μg/ml, Wilfactin, Sanquin, the Netherlands) was used to create a reference curve. Values for VWF levels in platelet lysates were corrected for total platelet protein content using Bradford analysis. The investigator performing the assay was blind to the samples' genotype.

CD9 measurement on platelet surface

The surface expression of CD9 was measured, by using flow cytometry, in PRP of 365 healthy subjects, part of the Cambridge Platelet Function Cohort, by investigators blind to the subjects' genotype. PRP was generated by centrifuging whole blood at 250xg for 15 minutes and adjusted to a concentration of 5×10^6 platelets per ml in PBS with 0.32% trisodium citrate and 0.25% bovine serum albumin (BSA). Platelets were labelled using a FITC conjugated monoclonal antibody for CD9 (ALB6, Beckman Coulter) for 30 minutes. Samples were washed and analysed using an Epics XL Flow Cytometer (Beckman Coulter). The median fluorescence intensity (MFI) of the FITC-CD9 was obtained using Kaluza analysis software (Beckman Coulter). Mean platelet volume (MPV) was measured using a Sysmex Haematology Analyser (XE-5000).
