# Supplementary material for: Platelet function is modified by common sequence variation in megakaryocyte super enhancers
Source: Nat Commun. 2017 Jul 13;8:16058. doi: 10.1038/ncomms16058 (PMC5511350; doi:10.1038/ncomms16058)
Supplement: Peer Review File [file ncomms16058-s12.pdf]

Editorial Note: this manuscript has been previously reviewed at another journal that is not operating a transparent peer review scheme. This document only contains reviewer comments and rebuttal letters for versions considered at Nature Communications.
